# Enhanced control of *Mycobacterium tuberculosis* extrapulmonary dissemination in mice by an arabinomannan-protein conjugate vaccine

**DOI:** 10.1371/journal.ppat.1006250

**Published:** 2017-03-09

**Authors:** Rafael Prados-Rosales, Leandro Carreño, Tingting Cheng, Caroline Blanc, Brian Weinrick, Adel Malek, Todd L. Lowary, Andres Baena, Maju Joe, Yu Bai, Rainer Kalscheuer, Ana Batista-Gonzalez, Noemi A. Saavedra, Leticia Sampedro, Julen Tomás, Juan Anguita, Shang-Cheng Hung, Ashish Tripathi, Jiayong Xu, Aharona Glatman-Freedman, Williams R. Jacobs, John Chan, Steven A. Porcelli, Jacqueline M. Achkar, Arturo Casadevall

**Affiliations:** 1 Department of Microbiology and Immunology, Albert Einstein College of Medicine, Bronx NY, United States of America; 2 CIC bioGUNE, Bizkaia Technology Park, Derio, Bizkaia, Spain; 3 Millennium Institute on Immunology and Immunotherapy, Programa Disciplinario de Inmunologia, Facultad de Medicina, Universidad de Chile, Santiago, Chile; 4 Department of Medicine, Albert Einstein College of Medicine, Bronx NY, United States of America; 5 Howard Hughes Medical Institute, Albert Einstein College of Medicine, Bronx NY, United States of America; 6 Alberta Glycomics Centre and Department of Chemistry, University of Alberta, Gunning-Lemieux Chemistry Center, Edmonton, Alberta, Canada; 7 Grupo de Inmunologia Celular e inmunogenetica, Universidad de Antioquia, Medellin, Colombia; 8 Institute for Medical Microbiology and Hospital Hygiene, Heinrich-Heine-University Duesseldorf, Duesseldorf, Germany; 9 Ikerbasque, Basque Foundation for Science, Bilbao, Bizkaia, Spain; 10 Genomics Research Center, Academia Sinica, Section 2, Nankang, Taipei, Taiwan; 11 Infectious Diseases Unit, Israel Center for Disease Control, Israel Ministry of Health, Tel Hashomer, Israel; 12 Department of Pediatrics, and Department of Family and Community Medicine, New York Medical College, Valhalla, NY, United States of America; 13 Department of Molecular Microbiology and Immunology, Johns Hopkins Bloomberg School of Public Health, Baltimore, MD, United States of America; New Jersey Medical School, UNITED STATES

## Abstract

Currently there are a dozen or so of new vaccine candidates in clinical trials for prevention of tuberculosis (TB) and each formulation attempts to elicit protection by enhancement of cell-mediated immunity (CMI). In contrast, most approved vaccines against other bacterial pathogens are believed to mediate protection by eliciting antibody responses. However, it has been difficult to apply this formula to TB because of the difficulty in reliably eliciting protective antibodies. Here, we developed capsular polysaccharide conjugates by linking mycobacterial capsular arabinomannan (AM) to either Mtb Ag85b or *B*. *anthracis* protective antigen (PA). Further, we studied their immunogenicity by ELISA and AM glycan microarrays and protection efficacy in mice. Immunization with either Abg85b-AM or PA-AM conjugates elicited an AM-specific antibody response in mice. AM binding antibodies stimulated transcriptional changes in Mtb. Sera from AM conjugate immunized mice reacted against a broad spectrum of AM structural variants and specifically recognized arabinan fragments. Conjugate vaccine immunized mice infected with Mtb had lower bacterial numbers in lungs and spleen, and lived longer than control mice. These findings provide additional evidence that humoral immunity can contribute to protection against Mtb.

## Author summary

Vaccine design in the TB field has been driven by the imperative of attempting to elicit strong cell-mediated responses. However, in recent decades evidence has accumulated that humoral immunity can protect against many intracellular pathogens through numerous mechanisms. In this work, we demonstrate that immunization with mycobacterial capsular arabinomannan (AM) conjugates elicited responses that contributed to protection against Mtb infection. We developed two different conjugates including capsular AM linked to the Mtb related protein Ag85b or the Mtb unrelated PA from *B*. *anthracis* and found that immunization with AM conjugates elicited antibody populations with different specificities. These surface-specific antibodies could directly modify the transcriptional profile and metabolism of mycobacteria. In addition, we observed a prolonged survival and a reduction in bacterial numbers in lungs and spleen in mice immunized with Ag85b-AM conjugates after infection with Mtb and that the presence of AM-binding antibodies was associated with modest prolongation in survival and a marked reduction in mycobacterial dissemination. Finally, we show that AM is antigenically variable and could potentially form the basis for a serological characterization of mycobacteria based on serotypes.

## Introduction

*Mycobacterium tuberculosis* (Mtb), the causative agent of TB, can establish latent or progressive infection despite the presence of a fully functioning immune system. The capacity of Mtb to avoid immune-mediated clearance reflects a necessary association with the human host that has led to an evolved and coordinated program of immune evasion strategies, including interference with antigen presentation to prevent and/or alter the quality of T-cell responses [1]. There is strong evidence to suggest that the mycobacterial cell envelope is of key importance for survival in the host [2]. The mycobacterial envelope consists of three major components: the plasma membrane, the cell wall, and an outermost capsule [2]. Bacterial capsules are protective structures important for the interaction with and successful colonization of the host [3]. Toxic substances have recently been found in the mycobacterial capsule, suggesting the contribution of this compartment to mycobacterial pathogenesis [4].

The mycobacterial capsule is loosely attached to the surface and is mainly composed of proteins and polysaccharides [2]. The major surface exposed capsule polysaccharides are a 120 kDa glycogen-like α-glucan, a 15 kDa arabinomannan (AM) and a 4 kDa mannan [5]. Both AM and mannan are structurally related to lipoarabinomannan (LAM), the major lipopolysaccharide of the mycobacterial cell wall. LAM is also known for having biological effects during its interaction with host cells, including immunosuppression of T cell responses or interference with macrophage activation [6]. LAM and AM can each elicit high antibody responses in infected hosts [7]. A low antibody to LAM response in children with TB was associated with disseminated mycobacterial disease [8]. That report concluded that a weak antibody response to LAM and other mycobacterial antigens increased the likelihood of dissemination [8]. Presumably, antibodies can also contribute to the host defense against Mtb by promoting the clearance of LAM [9]. In fact, several reports on AM or LAM-binding monoclonal antibodies have established their capacity to contribute to the clearance of mycobacteria from the host [10, 11].

In 2014, there were an estimated 9.4 million new cases of TB and 1.5 million people died from TB, including 1.1 million deaths among HIV-negative individuals and 0.4 million among people who were HIV-positive [12]. Efforts to control the disease include the development of “point-of-care” tests, new TB drugs, the use of the Bacille Calmette-Guerin (BCG) vaccine and the development of new vaccines. Most of the new vaccine candidates against TB that have entered in clinical trials fall into one of the following groups: (I) live attenuated vaccines to replace BCG; (II) subunit vaccines to be given after initial BCG vaccination [13]; and (III) single immunodominant antigens, usually secreted, such as ESAT-6, Cfp10 and Ag85b along with other adjuvants [13]. These vaccine candidates were developed with the working assumption that immunity against TB relied solely on cellular defense mechanisms [14]. While there is no doubt that cell-mediated immunity is a major arm in the control of mycobacterial infection, there are now compelling data that certain antibodies are active against mycobacteria [9–11, 15].

In this study, we have generated two different polysaccharide conjugates made of capsular Mtb AM and proteins Ag85b (Ag85b-AM) from Mtb H37Rv and protective antigen (PA) (PA-AM) from *Bacillus anthracis*, aiming to create AM-specific humoral immunity prior to challenging mice with virulent mycobacteria via aerosol. Previous studies using similar approaches have shown that secreted AM or delipidated LAM-containing conjugates provided some protection against Mtb infection in mice, rabbit or guinea pigs [15, 16]. Here we report that capsular AM conjugates promote an AM-binding antibody response in mice that is associated with reduced bacterial numbers in lungs and spleen, and prolonged survival in immunized mice. Our study provides additional evidence for an important role for antibodies in protection against Mtb and suggests that polysaccharide antigens could be useful components of future vaccines to fight TB.

## Results

### Capsular arabinomannan conjugates

Mtb H37Rv was grown in minimal media without Tyloxapol, which is known to release capsule [4]. After 14 d cultures were harvested and an aliquot was submitted to transmission electron microscopy (TEM) analysis to examine bacterial cells for the presence of the capsule. An electron transparent zone was clearly visible surrounding Mtb cells (S1 Fig). Visualization of Mtb cells under the scanning electron microscope revealed that the capsule is a matrix composed of small spherical units of approximately 50 nm in diameter [17] (S1 Fig). Arabinomannan (AM) is a low molecular weight polysaccharide that can be recovered from the upper phase of a chloroform-methanol-water extraction step [18] and separated from the other low molecular weight polysaccharides after proteinase K treatment by size exclusion chromatography (S1 Fig). Three major peaks were obtained of molecular mass 20 kDa (peak a), 10 kDa (peak b) and 4 kDa (peak c). According to the glycosyl composition analysis of the pooled peaks, peak a consisted of two main glycosides, arabinose and mannose in a ratio (2:1). This result is consistent with mycobacterial capsular AM and other reported analysis in mycobacteria [5, 18]. In addition, only peak “a” showed binding to 9d8 an AM-specific monoclonal antibody (Mab) and not to 24c5, recognizing α-glucan (S1 Fig). AM, as many other polysaccharides are poorly immunogenic because they are T cell independent antigens; therefore, immunization with polysaccharides generally does not elicit IgG immune responses. We hypothesized that vaccine-induced AM antibodies had value in protecting against TB. For this, we conjugated Mtb capsular AM, corresponding to the purify fraction (peak a) to different protein carriers. We did not make conjugates to other fractions that did not correspond to AM, as that was not the scope of this work. We selected Ag85b as Mtb-related protein carrier to test whether inclusion of AM would boost its recognized protective properties as an immunogen. In addition, we also linked Mtb capsular AM to the protective antigen (PA) from *Bacillus anthracis* as non-Mtb related antigen to set up a system where AM-binding immunity could be evaluated in an exclusive way. We developed Ag85b-AM and PA-AM conjugates using the cyanylating reagent CDAP as previously described [19, 20]. The conjugate products were separated by size exclusion chromatography on a Sephacryl S-200 (GE healthcare) (S2 Fig) in PBS. Ag85b-AM and PA-AM conjugates showed a protein-polysaccharide molar ratio of 1:8 and 1:7, respectively, as determined by Bradford and the phenol-sulphuric acid assays.

### Antibody response of AM conjugates in mice

To test the immunological response of the different AM conjugates, mice were immunized with different amounts of Ag85b-AM or PA-AM conjugates (1, 5 and 10 μg) in 1% Alum. Alternatively, three different groups of mice received 1 μg of either Ag85b, PA or 10 μg of AM, also in 1% Alum. Each mouse was boosted twice every two weeks and serum samples were taken to determine the kinetics of specific antibodies (S3 Fig). No antibody response was detected in PBS, AM or 1% Alum alone-immunized mice. We determined that immunization with three doses of 10 ug of Ag85b-AM conjugate provided elevated levels of AM-specific Abs (1:3000) (S3 Fig). We believe that our regime of immunization provided sufficient and stable levels of Ag85b-specific T cells as it has been previously shown using similar immunization approaches [21].

**Fig 1:**
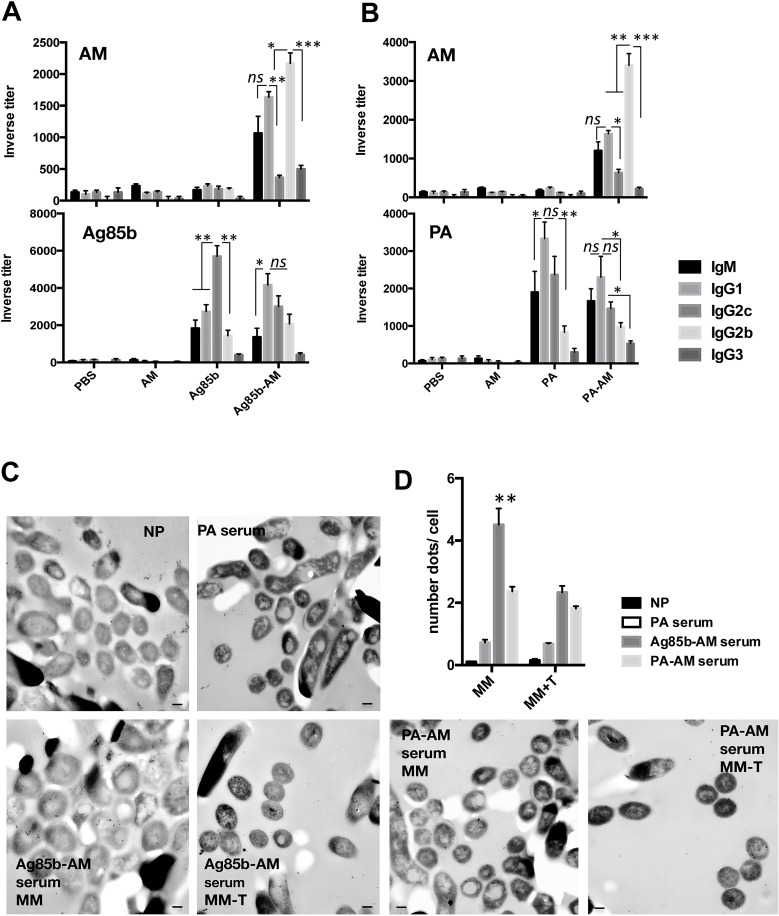
Antibody response to conjugate immunization in mice. **(A)** Titers of AM (Mtb) (top graph) or Ag85b (bottom graph)-specific antibodies measured by ELISA in serum from C57BL/6 mice (*n* = 3 per group) immunized with 10 μg of AM-Ag85b conjugate, 1 μg of Ag85b, 10 μg of AM or PBS. **(B)** Titers of AM (Mtb) (top graph) or PA (bottom graph)-specific antibodies measured by ELISA in serum from C57BL/6 mice (*n* = 3 per group) immunized with 10 μg of AM-PA conjugate, 1 μg of PA, 10 μg of AM or PBS. The results are representative of three independent experiments performed in the same manner. **(C)** Immunogold electron microscopy of thin sections of Mtb H37Rv cells treated with immune sera specific for the indicated antigens and detected with a 6-nm IgG gold-labeled anti-mouse antibody. Immunolabeling was tested in Mtb grown in minimal medium with (MM-T) and without tyloxapol (MM). Scale bars: 100 nm. **(D)** Quantitative analysis of the immunobeling of immune sera by determining the number of gold particles per cell. Bars are mean +/- sem. NP, denotes No Primary antibody. **P < 0.01 using one-way ANOVA. Data are mean +/- sem. *ns*, not significant.

We further analysed the IgG subclasses and IgM in sera isolated at day 45 after initial immunization (Fig 1A and 1B). Immunization with either Ag85b or PA induced high levels of protein-specific antibodies (1:6000, 1:4000) and no polysaccharide-specific antibodies as expected. Immunization with conjugates promoted an antibody response to both the protein and polysaccharide components of the conjugates, being the response to proteins very similar to that the immunization with protein alone. Ag85b-specific Ab response consisted on a mix of all subclasses being IgG2c the most prevalent with a three fold increase relative to the other groups. Immunization with PA alone induced a mix of IgM, IgG1 and IgG2c (Fig 1A and 1B). The AM-binding antibody response was very similar between the two conjugates in terms of diversity. A mix IgM, IgG1 and IgG2b was observed in AM-Ag85b immunized mice versus an exclusive IgG2b in AM-PA immunized mice.

Since AM is a mycobacterial capsular polysaccharide we considered whether AM polyclonal sera would recognize the outermost compartment of Mtb. However, any interpretation of the data needs to take into account the fact that AM and LAM share epitopes, suggesting that AM-immune serum might also label LAM. In fact, analysis of the specificity of AM-serum for binding to other Mtb cell wall components by ELISA revealed a cross reactivity with LAM, ManLAM and LM and absence of binding to arabinogalactan (AG), mycolyl-arabinogalactan-peptidoglycan complex (mAGP) or trehalose deoxy mycolate (TDM) (S4 Fig). Notably, there is no Ab available to distinguish between AM and LAM. To explore the location of Ab binding we utilized immunogold TEM with AM-binding sera (Fig 1C and 1D and S5 Fig). We used encapsulated Mtb cells that were generated by growing mycobacteria in the absence of detergent. It is known that supplementation of the culture with detergent strips the mycobacterial capsule [22]. Grids containing sections of Mtb cells were labelled without any primary antibody (NP) as controls and no immunogold was detected. Similarly, no labelling was observed when the experiment was performed with a PA-binding serum, confirming the lack of cross reactivity of PA-binding antibodies to Mtb. We observed labelling in all conditions where AM-binding sera were used. The location of the immunogold particles in cells labelled with Ag85b-AM serum was not restricted to the surface but distributed all over the bacterial cell (Fig 1C). On the contrary, most of the immunogold labelling observed in grids treated with AM-PA serum was restricted to the bacterial surface. Since both conjugates were generated with the same AM molecule, surface labelling is most probably due to AM or LAM. However, in the case of Ag85b-AM-immune serum, Abs to Ag85b could also be labelling this protein throughout the mycobacterial cell, explaining the broad distribution of the labelling that we observed.

When immunolabeling was performed on grids containing unencapsulated mycobacteria (grown in the presence of detergent) we observed a reduction on labelling, indicating that most of the material being recognized in encapsulated Mtb is not present in unencapsulated Mtb (Fig 1C and 1D). These results indicate that immunization with AM-conjugates induces a potent and specific antibody response primarily directed to the mycobacterial surface and specifically to the capsule.

### Dissection of the AM-binding antibody response by glycan microarrays

**Fig 2:**
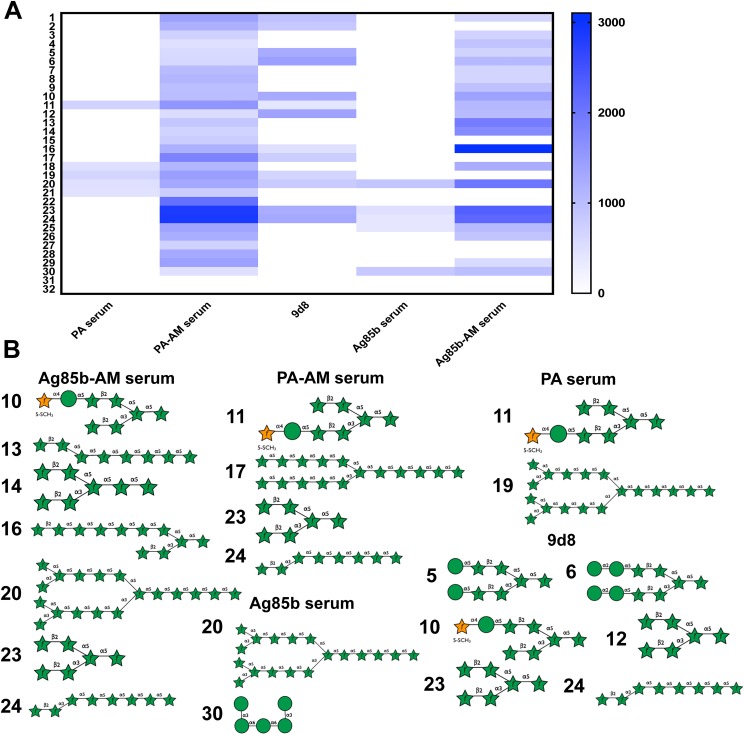
Assessment of the relative IgG-binding of AM immune sera the 29 printed AM fragments. **(A)** Heat map of AM glycan microarray data obtained after incubation with pooled murine sera diluted 1:400 followed by labeled anti-IgG. Data are mean of three independent spots. Values are relative fluorescence units. **(B)** AM fragments included in the glycan microarray representing the AM molecule recognized by the indicated serum. The numbers correspond to those in A and S6 Fig.

AM is a neutral and heterogeneous capsular polysaccharide comprised of a mannan backbone substituted by a branched arabinan, further modified by mannose residues at the non-reducing end [23]. To gain insight into the specific differences in serum reactivity provided by the conjugates, we analyzed immune sera on glycan microarrays including 30 synthetic AM fragments (Fig 2 and S6 Fig). The synthetic AM fragments included on the array are representative of the motifs present in all three of these domains (S6 Fig). Selection of compounds for synthesis was based on the reported structure of AM.

Further, AM-arrays were probed with diluted sera from either AM-PA or AM-Ag85b conjugate-immunized mice. PA and Ag85b-immunized mice and the AM-binding monoclonal antibody (mAb) 9d8 were used as controls. We observed a more diverse repertoire of AM fragment recognized by conjugate sera relative to the control mAb 9d8 (Fig 2A). This is consistent with the response expected from a polyclonal serum versus a mAb. A reduced response was detected in arrays probed with the PA or Ag85b-serum for the majority of the epitopes. Notably, we observed a common reactivity profile between sera from both conjugate-immunize mice, indicating that the conjugated PS might have been modified similarly. More specifically, we observed a prevalent recognition for epitopes ranging from linear arabinose fragments including 4 to 8 sugar units (#24) to highly branched arabinose polysaccharides (#16, #17, #20, #23 and #24). (Fig 2A and 2B). The highest reactivity in AM-PA serum was observed for fragments 23, 24 and 22, whereas in AM-Ag85b serum was observed for fragments 16, 23, 24 and 20. All fragments represented linear or branched arabinose polysaccharides, except for fragment 22, which included the arabinan core of fragment 14 but highly mannosylated (three mannose residues) at both reducing ends. Fragments 5 and 6 were preferentially recognized by the mAb 9d8 and included structures with a short-branched arabinan core manosylated at both ends. Both conjugate sera shared reactivity of fragments 1, 10 and 12 with 9d8. These fragments included short and linear arabinose glycans (#1), short and branched arabinan molecules with low mannosylation (#12) and xylose-substituted at the mannose reducing end (#10). These results suggest that AM-binding antibody response is directed to a diverse set of glycans, mostly associated to the arabinan core and that the protective mAb 9d8 reacts to a reduced set of glycans including a less complex repertoire.

### Transcriptional response of Mtb during AM-binding antibody interaction

**Fig 3:**
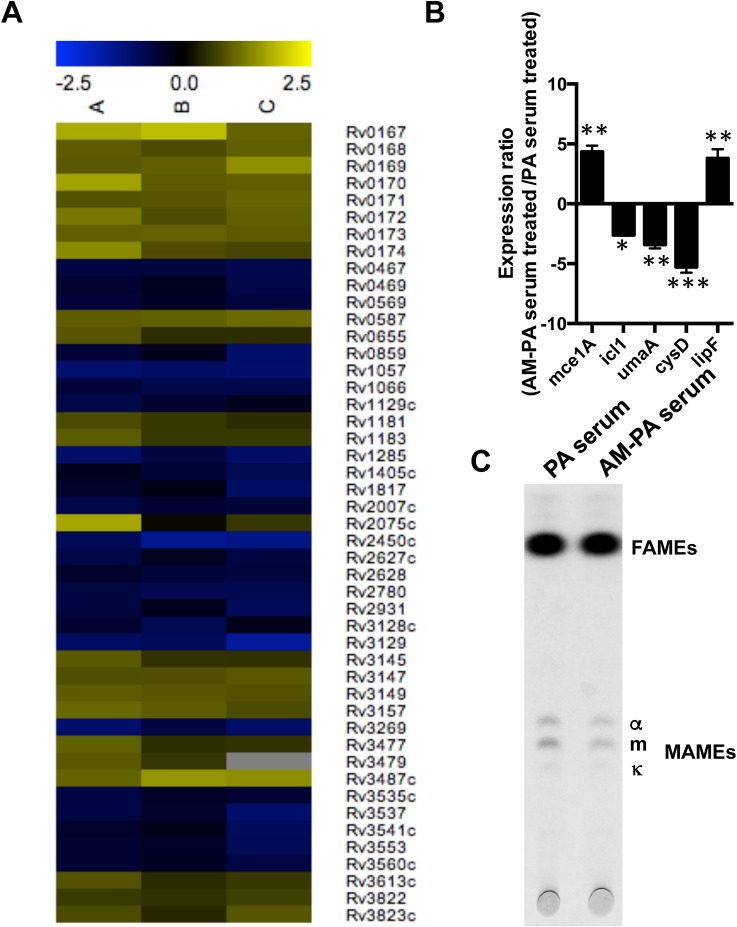
Effect of AM immune serum on the transcriptional profile of *M*. *tuberculosis*. **(A)** Transcriptomic profile of Mtb during treatment with AM-PA murine serum (1:400) compared to PA murine serum (1:400) for 4 h. The heat map shows transcriptional changes from three biologically independent replicates labelled as A, B and C. **(B)** Expression ratio of the indicated Mtb genes measured as the average relative expression of AM-PA serum vs PA serum-treated Mtb by quantitative real time PCR (qRT-PCR). (*P < 0.05, **P<0.01 ***P < 0.001, one-way ANOVA with Tukey post-test). **(C)** Analysis of fatty acid methyl esters (FAMEs) and mycolic acid methyl esters (MAMEs) in *M*. *tuberculosis* H37Rv labelled with ^14^C-acetate for 22h prior to treatment with the indicated serum preparations for 5 h. Lipids were extracted and analyzed by TLC as described in Methods. The same amount of cpm was spotted for each sample.

Recently a new function for humoral immunity was described whereby the binding of specific antibodies to microbes triggered transcriptional responses that were associated with physiological changes [24, 25]. Consequently, we investigated whether capsular AM-binding antibodies elicited transcriptional changes in Mtb by incubating encapsulated Mtb with AM-PA serum for 4 h and comparing the changes in transcription with a condition including PA immune serum using microarrays (Fig 3). Microarray data was deposited with the GEO NCBI database with the accession number GSE77711.

We observed a consistent and significant upregulation of most of the *mce1* operon (Fig 3A and 3B), consisting of 13 genes encoding a putative ABC lipid transport system specialized in mycolic acids [26]. Upregulation of the *mce1* operon was also observed when Mtb is inside host cells [27]. Moreover, an Mtb mutant defective in the *mce1* operon was shown to be hypervirulent in mice and produce more mycolic acids [28, 29]. The fact that *umA*, a mycolic acid synthase, was downregulated lead us to hypothesize that upregulation of *mce1* operon could result in a reduction in the mycolic acid content of Mtb cells upon interaction with AM-binding immune serum. Indeed, when we measured fatty acids by TLC, we observed reduction in alpha, keto and methoxy mycolic acids levels (Fig 3C), establishing that antibody-induced transcriptional changes resulted in mycobacterial biochemistry changes.

We also observed upregulation in some of the *nuo* genes, which are involved in aerobic respiration (Fig 3A). Of note, the gene encoding for isocytrate lyase (*aceA*, *icl1*), which is known to be required for persistence in the mouse model, was downregulated in Mtb treated with AM-binding serum. Similarly, transcript levels of *cysD*, which encode a sulfate adenylyltransferase involved in sulphate metabolism, were reduced in AM-treated Mtb (Fig 3A and 3B). We could not explain why *lipF*, encoding a lipid esterase, appeared as downregulated in the microarray while this transcript showed a four-fold upregulation relative to untreated Mtb (Fig 3B). These results indicate that antiserum including antibodies with specificities for Mtb capsular AM can alter the lipid metabolism and the fitness of mycobacteria.

### Protective efficacy of AM conjugates in mice

**Fig 4:**
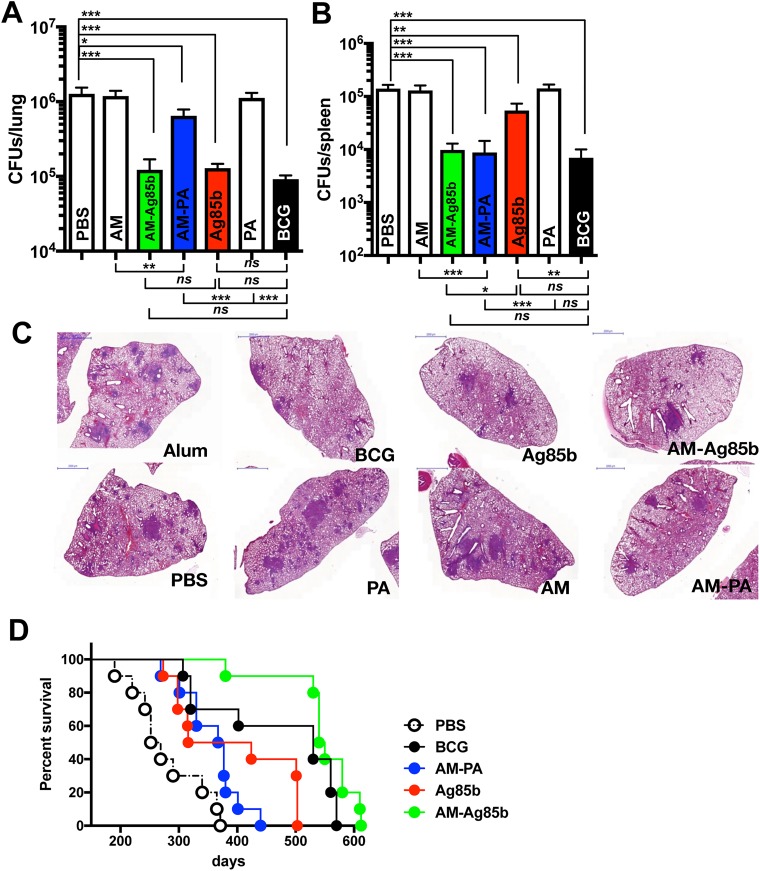
Immunization with conjugates protect against Mtb infection. **(A,B)** Bacterial load (CFUs) in the lungs **(A)** and spleen **(B)** of individual C57BL/6 mice, immunized with the indicated preparations was determined at 4 weeks after infection with a low dose of Mtb H37Rv via aerosol (approx. 100 CFUs). The results are pooled values from two similar and independent experiments. Experimental groups used 5 mice. (*P < 0.05, **P<0.01 ***P < 0.001, one-way ANOVA with Tukey post-test). **(C)** Representative H&E staining images from lungs of C57BL/6 mice immunized with the indicated preparations and aerosol infected with Mtb H37Rv for 4 weeks. A representative lung section for each treatment is shown. **(D)** Survival of mice immunized with PBS (*n* = 10), 1 × 10^6^ CFU of BCG (*n* = 10), 10 μg of AM (Mtb)-Ag85b (*n* = 10), 10 μg of AM (Mtb)-PA conjugate (*n* = 10) or 1 μg of Ag85b (*n* = 10) and challenged with ~100 CFU of aerosolized Mtb H37Rv. All the immunized mice were significantly different from that of PBS-treated mice (*P* < 0.001, log-rank test for AM-Ag85b and BCG; *P =* 0.0064, log-rank test for Ag85b). No differences between the immunized groups were found except for Ag85b vs AM-Ag85b immunized mice (*P* = 0.0166). The survival curve for AM(Mtb)-PA was significantly different from that of PBS (*P* = 0.049, Gehan-Breslow-Wilcoxon test; *P* = 0.057, log-rank test). Survival studies were performed twice with similar results.

To separately test the ability of the two conjugates (Ag85b-AM and PA-AM) to modify the course of Mtb infection, mice were immunized three times with 10 μg of each conjugate and challenged with virulent tubercle bacilli by the respiratory route 4 weeks after the last immunization. Immunization controls included AM, PBS (1% Alum), and 1 x 10^6^ BCG. At 4 weeks after challenge mice were sacrificed and bacterial loads were assessed in lung and spleen (Fig 4A and 4B). As Ag85b is a well known immunogenic and protective Mtb antigen [30], we initially tested whether AM-Ag85b conjugate could control bacterial replication more efficiently than Ag85b alone. An immunizing dose of Ag85b equivalent to that of included in the conjugate was used to generate Ag85b-immunized mice. Both conjugate and Ag85b-immunized mice showed similar reduction in mycobacterial numbers in the lung at 4 weeks (Fig 4A). Conversely, we noticed a more significant reduction in bacterial counts in spleen in AM-Ag85b immunized mice, similar to BCG-immunized mice (Fig 4B). Histological analysis revealed marked differences in tissue inflammation in mice immunized with AM, and adjuvant relative to those immunized with AM-Ag85b and Ag85b mice (Fig 4C), with the latter groups manifesting more intact lung morphology with less inflammation. We observed a major difference in the gross pathology of lungs from the AM-Ag85b-immunized mice compared to Ag85b-immunized mice, as evident by less diseased tissue. AM-Ag85b immunized mice showed a reduction in both the number of infiltrates and the percentage of diseased tissue, although these differences were not significant (S7 Fig). Next, we tested for the ability of these conjugate to influence the survival of mice challenged with a low dose of virulent tubercle bacilli via aerosol (Fig 4D). As a positive control, we included mice immunized with 1 x 10^6^ BCG. All immunized mice, including BCG, Ag85b and conjugate immunized mice significantly lived longer than the non-immunized mice injected with adjuvant. No differences were observed between BCG and conjugate immunized mice. Mice immunized with AM-Ag85b lived significantly longer than Ag85b immunized mice (*P* = 0.0166) indicating that AM-binding antibodies contributed to prolonging the survival of infected mice. These results suggested that antibodies to Ag85b were also protective and could be masking any protective contribution of AM-binding immunity against Mtb whereas the longer survival in AM-Ag85b immunized mice suggested that AM-binding antibodies contributed to protection. Consequently, we analyzed the protective efficacy of AM-PA conjugates to assess the exclusive contribution of AM-binding immunity. Of note, a 0.42 log reduction (*P* = 0.04) in lung CFUs was observed in mice immunized with AM-PA conjugates (Fig 4A). Immunization with either AM and PA alone did not provide any protection and similar CFUs numbers as in 1% Alum-treated mice were counted in the lungs. When we analyzed bacterial loads in spleen we observed a significant reduction in CFUs in AM-PA immunized mice, similar to AM-Ag85b and BCG immunized mice, followed by Ag85b (Fig 4B). Consistent with the relatively weak ability of AM-PA conjugates to control bacterial replication in the lungs, we observed comparable lung pathology to non-immunized or PA and adjuvant-immunized mice (Fig 4C). Although we measured a mean of 5 infiltrates in lungs of PA-AM immunized mice versus 8 infiltrates in adjuvant-immunized mice, these differences were not significant. However we did measure a significant reduction in the percentage of diseased tissue (S7 Fig). AM-PA conjugate-immunized mice lived longer than adjuvant-treated mice (median survival 337 days vs 297; log-rank *P* = 0.057, GBW *P* = 0.049) and mice receiving BCG as a vaccine showed a survival mean time of 479 days (p>0.001) (Fig 4D). These results suggest that immunity directed to AM can contribute to reduced bacterial dissemination and lung inflammation, which in turn translated into prolonged survival of infected mice.

### AM-binding antibodies contribute to control bacterial dissemination

**Fig 5:**
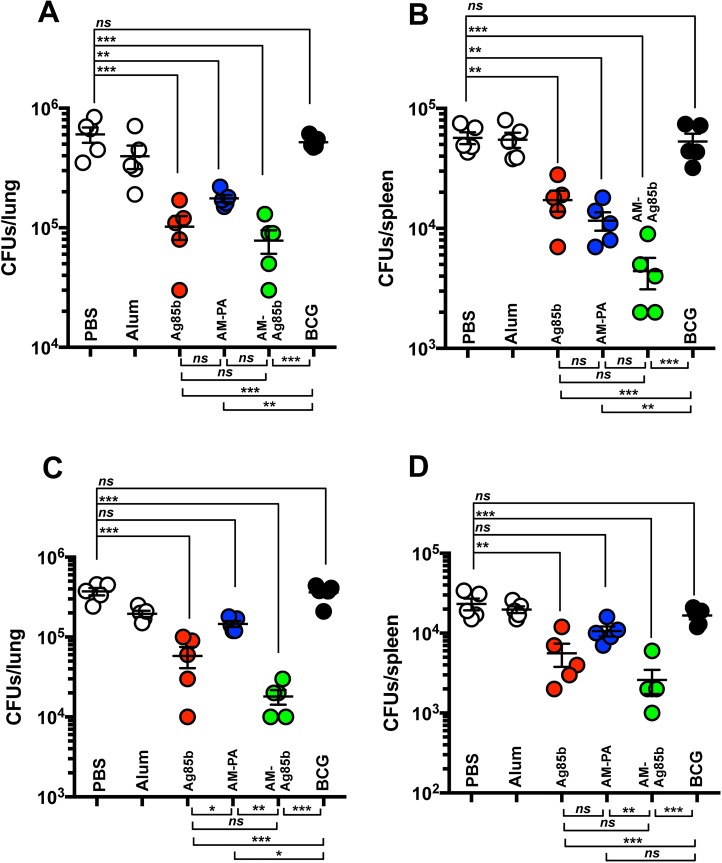
Passive transfer of immune serum and adoptive T cell transfer modify the course of mycobacterial infection. **(A,B)** Bacterial burden (CFUs) in the lungs **(A)** and spleen **(B)** at 4 weeks after infection with a low dose of Mtb H37Rv via aerosol (approx. 100 CFUs) of individual naïve C57BL/6 mice that previously received passively serum preparations. Results are representative of two similar and independent experiments. Experimental groups used 5 mice. (*P < 0.05, **P<0.01, one-way ANOVA with Tukey post-test). **(C,D)** Bacterial load (CFUs) in the lungs **(C)** and spleen **(D)** of individual naïve C57BL/6 mice adoptively transferred with 4 x 10^6^ T cells from the indicated immunized mice were determined at 4 weeks after infection with a low dose of Mtb H37Rv via aerosol (approx. 100 CFUs). The results are representative of two similar and independent experiments. Experimental groups used 5 mice. (*P < 0.05, **P<0.01, one-way ANOVA with Tukey post-test).

To establish that the protection observed following conjugate immunization was due to humoral immunity we carried out a passive antibody transfer experiment using sera from immunized mice. Mtb bacterial counts were enumerated in lungs and spleens 4 weeks after challenge with a low dose of Mtb via aerosol (Fig 5A and 5B). We found that passive administration of sera from Ag85b and AM-Ag85b-immunized mice was associated with reduced bacterial CFUs in lung, as compared to adjuvant and naïve (PBS) mice (Fig 5A). In mice that received AM-PA-immune serum, there was a significant reduction in lung CFU of 0.4 log relative to PBS and adjuvant. Of note, although we observed a greater reduction in lung CFUs in mice that received Ag85b, this did not reach statistical significance relative to mice transferred with PA-AM-serum. Consistent with the ability of AM-PA and AM-Ag85b conjugates to control bacterial dissemination (Fig 4B), we observed that sera from conjugate-immunized mice significantly reduced the bacterial CFUs in spleen, with AM-Ag85b-immune serum being superior to AM-PA-immune serum (no statistically significant differences, *P* = 0.054) (Fig 5B). We observed no benefit from the transfer of serum from BCG immunized mice. These results strongly indicate that specific antibodies to AM and Ag85b contribute to control bacterial dissemination. The greater protective efficacy achieved by AM-Ag85b immune serum might be due to either synergistic or additive effects of antibodies to these two antigens.

### T cells from AM-Ag85b mice mediate protection against *M*. *tuberculosis*

We next tested the ability of memory T cells from long-term immunized mice (S8 Fig) to protect naïve recipient mice against virulent Mtb by using a T-cell adoptive transfer protocol. Bacterial counts were enumerated in lungs and spleens 4 weeks after challenge with a low dose of Mtb via aerosol (Fig 5C and 5D). Remarkably, mice transferred with T cells from AM-Ag85b or Ag85b immunized mice showed significant reduction in CFU in the lung (Fig 5C) and spleen (Fig 5D), relative to PBS and Alum-mice. Mice receiving AM-PA-specific T cells did not show any capacity to control bacterial replication in lung and spleen (Fig 5C and 5D). The transfer of T cells from Alum or BCG-vaccinated mice did not translate into any protection. The later result is consistent with reports that transfer of BCG-induced immunity requires sublethal irradiation of recipients [31]. These data strongly suggests that the superior protection provided by AM-Ag85b conjugates is a combination of both AM and Ag85b-binding antibodies and Ag85b-specific T cells.

### Antigenic variability of capsular AM

**Fig 6:**
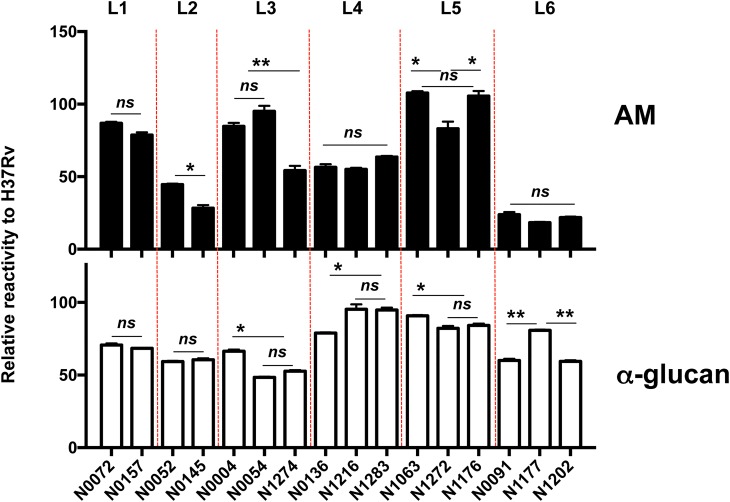
Antigenic variability of AM among Mtb isolates. Relative reactivity of Mtb isolates representing 6 of known lineages. The binding is shown as the percentage of reactivity compared to Mtb H37Rv. Data are mean +/- sem. Results are representative of two independent experiments. (*ns*, non significant, *P < 0.05, **P<0.01, one-way ANOVA with Tukey post-test).

Some encapsulated variants of many bacterial clinical isolates are associated with higher rates of mortality and morbidity and consequently vaccine development is focused on the these serogroups [32]. Carbohydrate antigens exhibit tremendous structural variation that can translate into antigenic variation as demonstrated by the 13 different serogroups of *Neisseria meningitidis*, over 90 different serotypes in *Streptococcus pneumoniae* or the more than 80 serotypes in *Klebsiella* sp [33]. However, for Mycobacterial spp. the question of polysaccharide-related antigenic differences on the bacterial surface has not been investigated. We hypothesized that AM presents antigenic variability among Mtb isolates given that it is a variable structure comprising a mannan backbone substituted by a branched arabinan, which is further modified by mannose residues at the non-reducing end. To investigate AM antigenic variability we analyzed a panel of 17 strains, including Mtb H37Rv, representing the 6 known lineages of Mtb strains globally distributed [34] and tested for AM and α-glucan reactivity to the 9d8 mAb (AM) and 24c5 mAb (α-glucan), respectively (Fig 6). We could measure some statistically differences in Ab recognition between isolates form the same lineage. However, we also could establish some correlates. We observed that AM of isolates from L1, L5 and some from L3 showed the highest binding to 9d8 mAb. L6 showed the lowest binding with a reduction of an 80% relative to Mtb H37Rv. Isolates from L4 showed a 50% reduction in AM binding to 9d8. Of note, we did not find as much variability in the binding of α-glucan to mAb among the different isolates. Most of the isolates showed a reduction in binding relative to Mtb H37Rv, in the range of 50–60%. Only isolates from L4 and L5 showed a similar reactivity. These results suggest that AM is the main source of antigenic variability in the mycobacterial capsule and establish different antigenic groups among Mtb clinical isolates. However, we cannot rule out that the relative distribution of capsular polysaccharides may be relevant for their binding to specific Abs. To understand the antigenic variability contributed by AM would require a more extensive analysis, including more Mtb isolates.

### AM-conjugate specific serum is opsonic to unencapsulated bacteria

Standard protocols for murine infection with Mtb use inoculum as a homogeneous bacterial suspension after growth in detergent. However, the generation of single cell suspensions using detergent to avoid clumping also can remove the capsular layer [2, 35]. Indeed, a recent study using Cryo-Electron microscopy (Cryo-EM) revealed that this layer is removed when cells are grown in the presence of detergent or agitation [4]. Since AM is part of the capsule we reasoned that capsular polysaccharide conjugate vaccines may be even more effective when the capsule is present and were concerned whether the bacteria in the inoculum would bind antibody since these had been treated with detergent. To test whether there was antigen on the surface of such cells we performed macrophage infections with Mtb previously opsonized with conjugate (AM-PA) serum and a preimmune mouse serum. Bacterial counts were enumerated 2 h after infection (S9 Fig). The percentage of bacterial uptake by macrophage was significantly higher when bacteria were previously treated with conjugate serum relative to an untreated control or bacteria treated with a preimmune serum. Notably, we could measure a statistically significant difference in the macrophage uptake of encapsulated Mtb relative to unencapsulated Mtb. This result indicates that the conjugate-specific serum still retain the opsonic properties even though the capsule may have been removed, at least in part, by growth in media with detergent.

## Discussion

Here, we demonstrate that immunization with mycobacterial capsular arabinomannan (AM) conjugates elicited responses that contribute to protection against Mtb infection. In this study we, (i) isolated capsular AM from the H37Rv strain of Mtb and developed conjugates with the Mtb related protein Ag85b and Mtb unrelated PA from *B*. *anthracis*; (ii) found that immunization with different AM conjugates elicited antibody populations with different specificities; (iii) showed that surface-specific antibodies could directly modify the transcriptional profile and metabolism of mycobacteria; (iv) observed a prolonged survival and a reduction in bacterial numbers in lungs and spleen in mice immunized with Ag85b-AM conjugates after infection with Mtb; (v) observed that the presence of AM-binding antibodies was associated with modest prolongation in survival and a marked reduction in mycobacterial dissemination; (vi) and demonstrated that AM is antigenically variable and could potentially form the basis for a serological characterization of mycobacteria based on serotypes.

Our conjugates generated antibodies to cell-surface AM and LAM, given the overlapping structural motifs in these glycoconjugates. It is noteworthy that the vaccinated mice were challenged with bacteria grown in the presence of detergent, a condition that strips the mycobacterial polysaccharide capsule [22], and thus reduces AM epitopes. Consequently, the efficacy of these conjugates may be greater against encapsulated bacteria.

To our knowledge, this study represents the first effort to investigate a native mycobacterial capsular polysaccharide in a vaccine against pulmonary Mtb infection. Prior studies evaluating AM antigens in experimental conjugate vaccines have used either secreted AM [16] or delipidated LAM [15]. In the former report, mice were immunized with extracellular AM conjugates including the recombinant *Pseudomonas aeruginosa* exoprotein A (rEPA), with no adjuvant, and challenged intravenously with *M*. *tuberculosis* Erdman. A moderate reduction in lung CFU was observed early in the course of infection [16]. In the latter, AM oligosaccharides (AMOs) derived from LAM of Mtb H37Rv were isolated and covalently conjugated to tetanus toxoid (TT) or to Ag85b from Mtb. Ag85b conjugates were given to mice in Alum providing significant protection compared to sham immunized mice as estimated by long term survival against an intravenous challenge Mtb H37Rv [15]. AMOs-TT conjugates were given subcutaneously followed by nasal boost in the Eurocine L3 adjuvant providing a similar level of protection after a similar challenge with virulent *M*. *tuberculosis*. Remarkably, the protective efficacy was comparable to that of BCG vaccine. In guinea pigs, immunization with AMOs-Ag85b in Eurocine L3 adjuvant followed by an aerosol challenge with Mtb H37Rv showed an increased in survival and reduced pathology in lungs and spleens relative to non-immunized animals [15]. It is difficult to compare the efficacy of these vaccines as each was tested using different conditions that included the route of immunization, mode of infective challenge or animal model. In fact, only the study using AMOs-Ag85b immunization followed by an aerosol challenge [15] can be compared to our study even though it differs in the mouse strain used as experimental host. Nevertheless, each of these studies provide the consistent result that antibodies to AM modified the course of Mtb infection to the benefit of the host.

Animals immunized with the AM-Ag85b conjugate lived significantly longer than Ag85b-immunized mice, indicating that AM-specific immunity contributes to the protection. Also, we found a similar reduction in CFUs between AM-Ag85b conjugate- and Ag85b-immunized mice. Because Ag85b is a highly immunogenic antigen from Mtb that can elicit protective responses, it is difficult to assess the contribution of AM-specific immunity to the overall protection. Consequently, we also developed AM conjugates including the Mtb unrelated protein PA to study the specific contribution of antibodies to AM to the overall protection and found that circulating antibodies against AM reduced mycobacterial extrapulmonary dissemination in mice, as significant fewer CFUs were detected in spleens. Moreover, mice immunized with AM-PA conjugates manifested only a modest prolongation in survival compared to adjuvant-immunized mice. That the effect on CFU was antibody mediated was confirmed by passively transferring AM-specific serum to naïve mice and showing a similar reduction in CFUs in spleen after infection.

Considering that only zwtterionic polysaccharides can elicit T cell responses [36] and that AM is a neutral polysaccharide, it is extremely unlikely that a polysaccharide-specific T cell response was responsible for the protective efficacy of the AM-PA conjugate. This fact is consistent with the finding that the transfer of AM-PA T cells does not confer any protection. We observed a trend to a superior protection of AM-Ag85b T cells relative to Ag85b-T cells. Although we do not have a clear explanation for this result we cannot rule out the possibility that the covalent conjugation of AM to Ag85b altered the processing of T cell epitopes in Ag85b to elicit enhanced T cell responses that could be translated into a better protection. A precedent for such an effect was reported in pneumococcal polysaccharide-protein conjugates where the type of polysaccharide altered the T cell response to protein epitopes [37]. Furthermore, conjugation of a PstS1 synthetic peptide to a branched polysaccharide, including a polylysine backbone and DL-alanine side chains elongated by glutamic acid, enhanced human T cell proliferation to peptide [38]. Cross-protection form heterologous unrelated antigen (heterologous immunity) has been reported in viral infection [39]. Experiments addressing the quality of Ag85b-specific responses, possibly involving tetramer analysis, will be of paramount importance to gain insight into this finding.

Our findings are consistent with and supportive of the view that antibodies can play a significant role in the overall protection against Mtb. In addition to the effects observed with antibodies to AM, we obtained evidence that Ag85b-specific antibodies contributed to the control of bacterial replication in lung and spleen as demonstrated by passive transfer experiments. The fact that bacterial numbers in spleens of mice transferred with AM-Ag85b-specific serum were lower relative to mice transferred with Ag85b-specific serum or AM-PA-specific serum, suggests a synergistic effect of antibodies to both the mycobacterial polysaccharide and protein conjugate components. It is noteworthy that Ag85b immunization was previously shown to induce protective responses against Mtb that were believed to be dependent only on cell-mediated immunity [40–44]. Our observations suggest that humoral responses to Ag85b could have also contributed to protection in those vaccine studies.

The specificity of antibodies elicited by AM conjugates was characterized using an array including a set of 30 fragments representing the AM molecule. The AM glycan microarray revealed an enhanced and more diverse response in mice immunized with Mtb AM conjugates than the AM-specific mAb 9d8. Surprisingly, the most reactive fragments included non-mannosylated arabinnan structures with variable grades of branching, indicating that the glycosydic determinant of AM antigenicity is the arabinnan and not mannose part. We observed weak binding of pooled sera from PA and Ag85b-immunized mice to some fragments. Although this binding was low and close to the limit of detection, we cannot rule out that these antigens elicited some weak cross-reactive antibody responses. The fact that AM might be antigenically variable suggests that the abundance or the features of this polysaccharide may be different from strain to strain. In fact, our finding that the reactivity of AM containing sera differed from strain to strain suggests a previously unknown antigenic variation at the mycobacterial surface. This result could have very important implications for vaccine design because it suggests the possibility that some of the variability of BCG and live vaccine candidates may have been the result of antigenic differences between immunizing and infecting strains. Furthermore, this antigenic variation implies that any vaccine attempting to target mycobacterial polysaccharides should include cocktails of polysaccharide or oligosaccharides to cover likely epitopes. In fact, such an approach has proven very successful in vaccination against pneumococcus.

Incubation of AM with mycobacteria resulted in altered metabolism of Mtb implying a direct effect of antibody on bacterial cells. This phenomenon was previously reported with fungi [24] and Gram positive bacteria [25] and is now extended here to mycobacteria. Upregulation of almost the entire *mce1* operon upon AM-specific antibody indicates a very specific response to immunoglobulin binding on the surface. This operon is highly induced when Mtb is inside the host and a *mce1* mutant has more intracellular fatty acids, including mycolic acids [26, 27]. We found that upon upregulation of *mce1* operon, Mtb reduces the synthesis of mycolic acids. This finding raises the possibility that Mtb may become more susceptible to macrophages after interaction with AM-binding antibodies since mycolic acids strongly contribute to Mtb cell wall permeability and rigidity. The ability of AM-binding antibodies to modify Mtb metabolism opens a new door in our understanding of the potential of humoral immunity against mycobacteria. In addition, we have recently demonstrated that increased human serum IgG titers to capsular AM were associated with enhanced mycobacterial opsonophagocytosis, increased phagolysosomal fusion and intracellular growth reduction in human macrophages; data suggesting that these effects of antibodies to AM are FcR-mediated [45]. We thus hypothesize that some of the effects of the Abs induced by the AM conjugate vaccine are FcR-mediated, while some of the Mtb transcriptional changes could reflect a more direct growth reducing effect on Mtb. Functional *in vitro* and *in vivo* studies with monoclonal Abs are needed to further elucidate the specific effects by which Abs to AM and its oligosaccharide epitopes contribute to the defense against Mtb infection.

Vaccine design in the TB field has been driven by the imperative of attempting to elicit strong cell-mediated responsive while disregarding humoral immunity against Mtb. This view was fueled by overwhelming evidence for an important role for cell-mediated immunity while the role for humoral immunity was considered inconsistent at best. Further fueling this emphasis was the notion that since Mtb is an intracellular pathogen, immunoglobulins as extracellular molecules cannot reach this pathogen. However, that two-dimensional view has been supplanted by the realization that humoral immunity can protect against many intracellular pathogens through numerous mechanisms [46, 47]. Moreover, recent studies have shown that immunoglobulins can have direct effects on microbes including triggering signal transduction and modulating their physiology [48]. The difficulty of assigning a functional role for Abs against Mtb was recently uncovered as being due to the heterogeneity of the Ab response [49]. In addition, human Mtb-specific IgAs have superior capacity to inhibit Mtb growth than IgG antibodies [50], indicating that mucosal immunity should be highly considered. In this regard, we could not measure AM and Ag85b-specific IgAs in serum and BALs, suggesting that the lack of protection provided by the PA-AM conjugate in the lung might be due to the absence of this Isotype.

Although the mechanism of action of antibodies to AM has not been determined, there are a number of possibilities by which these antibodies can mediate protection based on precedents from other systems. AM-binding antibodies could: (i) promote the ingestion by phagocytic cells and increased intracellular killing through FcR-mediated phagocytosis [45, 51]; (ii) alter the inflammatory response thus reducing host-damaging effects on the immune response [52]; iii) promote the clearance of polysaccharide antigens from tissues thus removing immunomodulatory antigens with deleterious effects on the immune response; and/or iv) modulate the metabolism of Mtb to make it more susceptible to host defense mechanisms [53]. Vaccines that elicit AM-binding antibodies may have the potential to recruit humoral immunity for host defense, which could achieve synergy with cellular immune mechanisms.

In summary, our findings are consistent with role of AM-binding antibodies in defense against Mtb infection and suggest that vaccines that elicit both humoral and cell-mediated immunity may be more protective than those that elicit either. These data suggest that antibody-mediated immunity can make an important contribution to the outcome of mycobacterial infection and provide a new impetus for developing vaccines that harness this arm of the immune system.

## Methods

### Strains and reagents

*M*. *bovis* BCG Pasteur and Mtb H37Rv were grown in minimal medium (MM) [KH_2_PO_4_ 1 g/l, Na_2_HPO_4_ 2.5 g/l, asparagine 0.5 g/l, ferric ammonium citrate 50 mg/l, MgSO_4_×7 H_2_O 0.5 g/l, CaCl_2_ 0.5 mg/l, ZnSO_4_ 0.1 mg/l, 0.1% (v/v) glycerol, and with or without Tyloxapol 0.05% (v/v; Sigma), pH 7.0] or in Middlebrook 7H9 supplemented with 10% (v/v) OADC enrichment (Becton Dickinson Microbiology Systems, Spark, MD), 0.5% (v/v) glycerol and with or without Tyloxapol 0.05% (v/v) for 14 days in a 5% CO_2_ incubator at 37°C. Mtb lineages were a gift from Sebastien Gagneux. Mtb lineages were systematically grown in MM supplemented with 30 mM pyruvate.

Recombinant Ag85b was obtained from AERAS Tb vaccine Foundation (Rockville, MD). Recombinant PA (Protective Antigen from *Bacillus anthracis*) was obtained from David Axelrod Institute, Albany, NY). The 1-cyano-4-dimethylaminopyridinium tetrafluoroborate (CDAP) and the other reagents used during the conjugation reaction were purchased from Sigma. The CS-35 monoclonal antibody recognizing LAM and AM, was obtained from BEI resources (Manassas, VA). The monoclonal antibody 9d8 specifically recognizes mycobacterial capsular AM [10, 54]. The monoclonal antibody 24c5 specifically recognizes mycobacterial capsular α-glucan [55]. Alhydrogel was purchased from InvivoGen (San Diego, US).

### Polysaccharide isolation

Capsular polysaccharides were isolated as described, with some modifications [5, 18]. Briefly, cells were pelleted from cultures at 3,450 x g for 15 min at 4°C. Five mg of glass beads (4 mm, Fisher) per g of cells were added and the mixture was gently shaken by vortex for 1 min. A volume of 50 ml of distilled water was added per g of disrupted cells and centrifuged at 8000 x g for 10 min at 4°C. The supernatant was recovered, clarified in a 0.22 μm filter unit (Millipore) and lyophilized. To separate the capsular arabinomannan (AM) from the rest of capsular polysaccharides, the capsule residue was resuspended in 4 ml of distilled water and subjected to a chloroform:methanol:water extraction (1:1:0.9). The upper phase was recovered and incubated in a rotavapor at 40°C overnight. Proteinase K (Sigma) was added at 10 mg/ml in a 50 mM Tris-HCl pH 7.5, 10 mM CaCl_2_ buffer and incubated overnight at 37°C. The deproteinated solution was dialyzed for 3 d at 4°C in distilled water, lyophilized and chromatographed on a column (90 cm x 1.8 cm) of Bio-Gel P-10 (Bio-Rad) using 0.1 M NaCl in 0.1% acetic acid. Collected fractions of 4 ml were assayed for carbohydrate content by the phenol-sulfuric acid assay. Pooled fractions were dialyzed in water and lyophilized. The concentration of protein was determined on each isolation step by Bradford.

### Conjugates

Mycobacterial AM-PA and AM-Ag85b conjugates were prepared as described [19, 56]. Briefly, 6 mg of AM was dissolved in 1 ml of borate buffer pH 9.0 and 60 μl of 100 mg/ml 1-cyano-4-dimethylaminopyridinium tetrafluoroborate (CDAP) was added and mixed with agitation for 10 min at room temperature. Then 6 mg of recombinant PA or Ag85b was added in 0.5 ml of 0.15 M HEPES pH 7.5, and the mixture was incubated for 1 h. The reaction was stopped with 100 μl of 0.15 M ethanolamine and incubated for 1 h. The mixture was then dialyzed in PBS for 2 days. To isolate the conjugates from the non-bound AM and PA or Ag85b, the dialyzed conjugated reaction was separated by FPLC on a Sephacryl SH200 (GE Healthcare) in PBS. Each fraction was then assayed for polysaccharide and protein content by the phenol-sulfuric acid assay and Bradford assay, respectively.

### Immunization

C57BL/6 female mice between 6 to 8 weeks old were purchased from Jackson Laboratories (Bar Harbor, MN). Animals were maintained in a specific pathogen-free animal facility under animal biosafety level-2 conditions for all experiments except for those involving infection with virulent Mtb for which animal biosafety level-3 conditions were used. Animals were immunized intraperitoneally (i.p) three times with 10 μg of Ag85b-AM or PA-AM conjugates including 1% (w/v) Alum, 1 μg of Ag85b or PA alone including 1% (w/v) Alum. Immunizations were given every two weeks. Control mice received i.p. injections of PBS or 10 μg of AM including 1(w/v) Alum. Alternatively, mice were vaccinated subcutaneously with 1 million BCG as a positive control in protection efficacy experiments or survival studies.

### Murine infections

Aerogenic challenge was done using a whole-body exposure aerosol chamber (Mechanical Engineering Workshop) custom fitted to a class III biosafety cabinet (Baker) to deliver approximately 100 CFU per animal of Mtb (H37Rv). Immunized mice were infected four weeks after the last immunization and eight weeks after BCG immunization. Mice were euthanized at 15 and 30 d after challenge. Lungs of individual mice were aseptically removed and homogenized separately in 5 ml normal saline plus 0.05% Tyloxapol using a Seward Stomacher 80 blender (Tekmar). The homogenates were diluted serially and plated on Middlebrook 7H11 agar to determine CFU of Mtb. Dilutions 10^−2^ and 10^−3^, and 10^−2^ and 10^−1^ were platted when counting CFUs in lungs and spleens, respectively.

In bacterial loads studies, animals infected with Mtb H37Rv were observed at least twice daily until they died or became moribund and were euthanized.

### Macrophage infection

J774 macrophages (ATCC TIB 67) were plated in 96-well plates in complete DMEM. The cells were washed with DMEM and infected with Mtb, previously grown in MM with or without Tyloxapol, at an MOI of 10:1 for 2 h at 37°C. Before infection bacteria were opsonized with 20 μg/ml of pre-immune serum or H37Rv-conjugate serum for 30 min. Cell lysates were prepared by removing the medium and lysing with 0.05% SDS. Serial dilutions of the lysate were plated on 7H11 agar, and incubated at 37°C for 21 days before counting CFUs.

### Transmission electron microscopy

Cells of *M*. *bovis* BCG Pasteur and *M. tuberculosis* H37Rv were grown in minimal medium and fixed with 2% glutaraldehyde in 0.1 M cacodylate at room temperature for 2 h, and then incubated overnight in 4% formaldehyde, 1% glutaraldehyde, and 0.1% PBS. After fixation the samples were stained for 90 min in 2% osmium tetraoxide, then serially dehydrated in ethanol and embedded in Spurrs epoxy resin. Thin sections were obtained on an Ultracut UCT (Reichert) and stained with 0.5% uranyl acetate and 0.5% lead citrate (Reichart, Depew, NY, USA). Immunogold TEM (IEM) was performed as previously described with a polyclonal murine immune serum diluted 1:300 or monoclonal murine IgG2a 9d8 antibody (10 μg ml^-1^) and then immunogold stained using 6 nm goat α-mouse gold (Electron Microscopy Sciences). Samples were viewed on a JEOL 100CXII or JEOL 1200EX at 80kV.

### Scanning electron microscopy

Cells of *M. bovis* BCG Pasteur and *M*. *tuberculosis* H37Rv were fixed with 2.5% glutaraldehyde, 0.1 M sodium cacodylate, 0.2 M sucrose, 5 mM MgCl_2_ pH 7.4 and dehydrated through a graded series of ethanol solutions. Critical point dry was accessed using liquid carbon dioxide in a Toumisis Samdri 795 Critical Point drier (Rockville,MD, USA). Sputter was coated with gold-palladium in a Denton Vacuum Desk-2 Sputter Coater (Cherry Hill, NJ, USA). Samples were examined in a Zeiss Supra Field Emission Scanning Electron Microscope (Carl Zeiss Microscopy, LLC North America), using an accelerating voltage of 5 kV.

### ELISA

Two types of ELISA were used in this study. In one assay polystyrene microtiter plate wells were coated with 50 μl of AM (5 μg/ml) in carbonate buffer (0.015 M Na_2_CO_3_, 0.035 M NaHCO_3_, 0.003 M NaN_3_; pH 9.8) or with 1 μg of recombinant proteins (PA or Ag85b) in PBS by incubating the plates 2 h at room temperature. The wells were then blocked by adding 200 μl of 2% BSA in TBS and incubated at 37°C for 1 h. Serum from conjugate-immunized mice, PBS-injected mice or recombinant Ag85b or PA-injected mice were added to the wells and incubated for 1 h at 37°C. The plates were then washed and 50 μl of a 1 μg/ml solution of Goat anti-mouse-alkaline phosphatase (GAM-AP) IgG1, IgG2b, IgG2c, IgG3 or IgM antibody (Southern Biotechnologies) for 1 h at 37°C. The ELISA plates were washed and developed by using *p*-nitrophenylphosphate substrate. Results are presented as inverse titers, what means the inverse of the greatest dilution that still gives a positive result, after removing the background (2 times OD from control serum). Isolated AM from KZN clinical isolates was also tested for reactivity to mAb 9d8 and AM-immune serum following the above protocol.

Whole cell ELISA was used to measure the relative reactivity of monoclonals Ab 9d8 and 24c5 to different clinical isolates. For this ELISA, mycobacterial cells were killed by heating to 80°C for 2 h. Bacterial cells were dispersed by drawing up and expelling the bacterial suspension 10 times through a 25-gauge needle attached to a 1-ml syringe. The suspension was then allowed to settle in a transparent 1.5-ml microcentrifuge tube, and the supernatant was removed, leaving 100 μl of settled bacteria. The number of bacteria used was standardized according to the amount of protein in a 100-μl volume of sedimented bacteria. Coated plates were blocked as above and incubated with either a-glucan-specific mAb 24c5 or AM-specific mAb 9d8. The plates were then washed and 50 μl of a 1 μg/ml solution of goat anti-mouse-alkaline phosphatase (GAM-AP) IgG antibody was added to each well for 1 h at 37°C. The ELISA plates were washed and developed by using *p*-nitrophenylphosphate substrate.

### AM microarray

A panel of 30 AM fragments (corresponding to motifs at the non-reducing terminus of the molecule, which have previously been shown to be recognized by anti-AM/LAM Abs) [57–59] were synthesized, and coupled to BSA via a squarate-linker [60]. Arrays were printed and used as described [61]. Briefly, after blocking with 3% BSA/PBS, AM microarrays were incubated with diluted mouse sera (1:400), or the murine IgG2a mAb 9d8 (known to recognize only Mtb AM) [62], followed by incubation with goat anti-mouse biotin-labeled IgG (Southern Biotech, AL; Jackson Immunoresearch, PA) and incubation with a Streptavidin probe tagged with SureLight-P3 Cy5 (Cayman Chemicals, MI). The slides were scanned using the GenePix 4000 Microarray scanner system (Molecular Devices, CA). Images were analyzed by the image-processing software Spotfinder (http://www.tm4.org/spotfinder.html), which measured median pixel intensity (MPI) and neighboring background pixel intensity (BPI) of individual spots. The median fluorescent reactivity (MFI), representing AM-epitope specific Ab responses, was the MPI subtracted by the BPI. The minimum value of pixel intensity was determined by the MFI of the spots with low quality, which was determined by the software quality control score for each spot depending on signal-to-noise ratio and spot shape. The final MFI was averaged from the triplicates. The symbolic nomenclature used is that recommended by the Consortium for Functional Glycomics. Green circles = mannose; Green stars = arabinose; orange stars = 5-thiomethyl-xylose; white ovals = inositol. The linkage position and stereochemistry between the monosaccharides is indicated over the line connecting them. 3P5 = a phosphate linkage between O3 of the inositol and O5 of an arabinose residue [63].

### Microarray analysis

*M*. *tuberculosis* was grown in MM with detergent for 6 days and then subcultured in fresh MM without detergent for 5 days. Cultures were harvested and submitted to a syringe and gentle sonication to breakdown the clumps. Bacterial cells were further incubated with AM-PA and PA immune sera (1:200 dilution) for 4 h. After treatment cell were washed once on PBS and resuspended in Trizol (Ambion, Carlsbad, CA). Cells were disrupted by mechanical lysis in a FastPrep-24 instrument (MP Biomedicals, Santa Ana, CA) in Lysing Matrix B tubes and RNA was purified with the Direct-zol RNA miniprep kit (Zymo Research, Irvine, CA). cDNA probes were prepared and hybridized to DNA microarrays (Microarrays, Inc. Huntsville, AL), which were scanned and analyzed as described previously [64]. Briefly, slides were scanned on a GenePix 4000A scanner (Molecular Devices, Sunnyvale, CA) and processed with the TM4 software suite (http://www.TM4.org). TIGR Spotfinder was used to grid and quantify spots. TIGR MIDAS was used for Lowess normalization, standard deviation regularization and in-slide replicate analysis, with all quality control flags on and one bad channel tolerance policy set to generous. Results were analysed in MeV with Significance Analysis of Microarrays (SAM) and hierarchical clustering algorithms. Microarray data was deposited with the GEO NCBI database with the accession number GSE77711.

For quantitative PCR (qPCR) experiments, diluted cDNA was used as a template at 50 ng per reaction for real-time PCR reactions containing primer sets designed by Primer 3 and SYBR Green PCR Master Mix (Applied Biosystems) in accordance with the manufacturers’ instructions. qRT-PCR reactions were performed on an ABI 9700HT real-time PCR cycler (Applied Biosystems).

### Fatty acid analysis

*M*. *tuberculosis* was grown in MM with detergent for 6 days and then subcultured in fresh MM without detergent for 5 days. Cultures were harvested and submitted to a syringe and gentle sonication to breakdown the clumps. Bacterial cells were further labeled with ^14^C-acetate (10μCi in 10ml culture) for 22h and incubated with AM-PA and PA immune sera (1:400 dilution) for 10 h. Bacterial pellets were treated with 20% tetrabutylammonium hydroxide at 100°C overnight. Cell suspensions were further methylated with methyl iodide (0.1ml) in dichloromethane (2ml) for 1h and the organic phase was washed twice and dried [65]. Fatty acids were analyzed by TLC (hexane/ethyl acetate; 95/5; 3 elutions).

### Histology

Lungs were removed and fixed in 10% neutral buffered formalin (Fisher Scientific, Fair Lawn, NJ). Tissues were embedded with paraffin, sectioned at 5 μm thickness, and stained with haematoxylin and eosin stain. Five different lung sections per mouse were analyzed. Slides were scanned with a Perkin Elmer P250 High Capacity Slide Scanner (Waltham, Massachusetts) at 2,000 dots per inch (dpi). Digitized images were then analyzed using ImageJ software to calculate the total disease area occupied by granuloma and the percentage of lung surface affected by pneumonia as well as the number of infiltrates per lung. The total disease area for the entire lung section was calculated by adding the values for each lesion. The total percentage of diseased tissue was calculated by dividing the total disease area by the entire lung section and multiplying by 100, using image J software.

### Passive serum transfer experiments

Blood was collected from the retro-orbital plexus from C57BL/6 mice immunized three times with either 10 μg of Ag85b-AM conjugate, 1 μg of Ag85b, 10 μg of PA-AM conjugate, 1 μg of PA in 200 μl of 1% (w/v) Alum. Vaccines were administered at two weeks intervals. Control sera were obtained from mice that received i.p. injections of PBS, 1% (w/v) Alum or 1 million of bacteria of BCG (subcutaneously). Sera were collected after clarification by centrifugation of clotted blood and stored at −80°C until use. Two hundred μl of immune and control sera were administered i.p. 4h before infection with 100 CFU of *M*. *tuberculosis* H37Rv. Four weeks after infection mice CFUs were assessed in lung and spleen.

### Adoptive T cell transfer experiments

Total T cell populations were isolated from spleens from C57BL/6 mice immunized three times with 10 μg of Ag85b-AM conjugate, 1 μg of Ag85b, 10 μg of PA-AM conjugate, 1 μg of PA in 200 μl of 1% (w/v) Alum. Control T cells were obtained from mice that received i.p. injections of PBS, 1% (w/v) Alum or 1 million of bacteria of BCG (subcutaneously). Spleens were homogenized and treated with RBC lysis buffer (Sigma–Aldrich, St. Louis, MO). Splenic T cells were purified using the Pan T cell isolation kit (Miltenyi Biotec, Germany). An aliquot of isolated T cells was stimulated with 1 μg ml^−1^ of the synthetic peptide antigens (Invitrogen): FQDAYNAAGGHNAVF (Ag85B-P25; residues 240–254 of MTb/BCG Ag85B, I-Ab restricted); and 5 μg ml-1 of PA from *Bacilllus anthracis* to assess their specificity. Unstimulated wells served as negative controls in naive mice. Samples were combined with 1 μg ml^−1^ soluble antibody to mouse CD28 (clone 37.51; eBioscience) and 1 μg ml^−1^ soluble antibody to mouse CD49d (clone 9F10; eBioscience). After 2 h at 37°C, 10 μg ml^−1^ of brefeldin A (Sigma) 10 μg ml^−1^ of monensin (Sigma) were added to all samples, followed by incubation for 6 h at 37°C. Cells were stained with blue LIVE/DEAD viability dye (Invitrogen) followed by antibody to FcγRII/III (clone 2.4G2; American Type Culture Collection), with fluorochrome-conjugated monoclonal antibodies for surface staining: antibody to CD3ε (clone 145-2C11; eBioscience), antibody to CD8α (clone 53–6.7; BD Bioscience), antibody to CD4 (clone GK1.5; BD Bioscience), and antibody to CD45R (B220) (clone RA3-6B2; BD Bioscience). Cells were fixed with 2% (vol/vol) paraformaldehyde, washed with permeabilization buffer (PBS with 1 mM Ca^2+^, 1 mM Mg^2+^, 1 mM HEPES [N-2-hydroxyethylpiperazine-N′-2-ethanesulfonic acid], 2% [vol/vol] FCS, and 0.1% [wt/vol] saponin) and then blocked in permeabilization buffer plus 5% (vol/vol) normal mouse serum (Jackson ImmunoResearch Laboratories). Intracellular cytokines were detected with fluorochrome-conjugated antibodies to IL-2 (clone JES6-5H4; eBioscience), IFN-γ (clone XMG1.2), TNF-α (MP6-XT22) (both from BD Biosciences). Data were acquired on an LSR II flow cytometer (BD Biosciences), and data analysis was performed using FlowJo software v.10 (Tree Star).

C57BL/6 mice were injected i.p with 4 mg/mouse of cyclophosphamide to partially deplete lymphocytes and promote engraftment of transferred cells [66], and 2 days later received adoptive transfer of 4 × 10^6^ isolated total T cells. Twenty-four h later the recipient mice were subjected to a low dose (50–100 CFU) aerosol challenge with Mtb H37Rv. Lungs and spleens were harvested for CFU counts 4 weeks after infection.

### Statistical analysis

Standard one-way ANOVA followed by Tukey’s multiple comparison test of the means was used to determine statistical significance of immune responses and protective efficacies of the conjugates. *P<*0.05 was considered statistically significant.

Survival data were analyzed by comparing Kaplan-Meier survival curves with a log-rank test (GraphPad Prism); after the log-rank test, a Grehan-Breslow-Wilcoxon modification of the log-rank test was used in an exploratory manner to apply more weight to early events in experiments where larger differences in early survival were observed.

### Ethics statement

Mouse studies were performed in accordance to National Institutes of Health guidelines using recommendations in the Guide for the Care and Use of Laboratory Animals. The protocols used in this study were approved by the Institutional Animal Care and Use Committee of Albert Einstein College of Medicine (Protocols #20120110; #20150110).

## Supporting information

S1 Fig Isolation of capsular AM. **(A)** Electron micrograph of Mtb H37Rv cells grown in minimal media without detergent. Notice the capsule surrounding the cells. Scale bar is 100 nm. **(B)** Scanning electron micrograph of Mtb H37Rv cells grown in minimal media without detergent. Arrow denotes the polysaccharide capsule. Scale bar 1 μm. **(C)** Gel chromatography of light Mtb capsular polysaccharides on a PD-10 size exclusion column. Fractions of 4 ml were taken and the carbohydrate content was estimated by phenol-sulphuric acid method. “Vo” means void volume. The pooled fractions are indicated by letters. **(D)** Binding of 9d8 (anti-AM) (top graph) and 24c5 (anti-α-glucan) (bottom graph) monoclonal antibodies at various concentrations of the indicated PD-10 fractions. The diagram indicates the ELISA configuration. PS, Polysaccharide fraction; AP, alkaline phosphatase; GAM, goat anti-mouse. Capsular polysaccharide isolation was performed up to four times using the same experimental conditions. The results are representative of three independent experiments.
(PDF)

S2 Fig Purification of AM-conjugates. **(A,B)** Separation of the conjugate reactions AM-Ag85b **(A)** or AM-PA **(B)** on Sephacryl S-200 size exclusion column in PBS. Fractions were monitored by on-line measurements of protein content at 280 nm (dotted line) and post-column by measurement of carbohydrate content (straight line) by phenol sulphuric acid method.
(PDF)

S3 Fig Kinetics of AM-binding antibodies after immunization with AM-Ag85b conjugates. Inverse titers (total IgG) of AM-binding antibodies measured by ELISA in serum from C57BL/6 mice (*n* = 3 per group) immunized with different amounts of AM-Ag85b conjugate. Mice were immunized every two weeks twice after initial immunization. Measurements were performed at 2, 4 and 8 weeks after the initial immunization.
(PDF)

S4 Fig Specificity of AM-immune serum. Inverse titers of Abs from AM-Ag85b conjugate serum for binding to different components of mycobacterial cell surface measured by ELISA in serum from C57BL/6 mice (*n* = 3 per group). Mice were immunized three times with 10 μg of AM-Ag85b conjugate. The results are representative of three independent experiments performed in the same manner. AM, arabinomannan; AG, arabinogalactan; LAM, lipoarabinomannan; LM, lipomannan; ManLAM, mannose capped LAM; TDM, trehalose deoxy mycolate; mAGP, mycolate arabinogalactan peptidoglycan. complex
(PDF)

S5 Fig Immunogold electron microscopy of thin sections of Mtb H37Rv cells treated with immune sera specific for the indicated antigens and detected with a 6-nm IgG gold-labeled anti-mouse antibody. Mtb cells were grown in the presence (MMT) or in the absence of detergent (MM). Scale bar 100 nm.
(PDF)

S6 Fig AM fragments included in the glycan microarray representing the AM molecule. The symbolic nomenclature used is that recommended by the Consortium for Functional Glycomics. Green circles = mannose; Green stars = arabinose; orange stars = 5-thiomethyl-xylose; white ovals = inositol. The linkage position and stereochemistry between the monosaccharides is indicated over the line connecting them. 3P5 = a phosphate linkage between O3 of the inositol and O5 of an arabinose residue [63].
(PDF)

S7 Fig Morphometric analysis of lung histopathology by assessing the number of infiltrates per lung (bottom graph) and the percentage of diseased tissue (top graph) (*P < 0.05, **P<0.01 ***P < 0.001, one-way ANOVA with Tukey post-test); *ns*, not significant. (PDF)

S8 Fig Specificity of the isolated T cells. Mice were immunized with AM conjugates, PA and Ag85b in Alum and after 4 weeks T cells were isolated. Specificity of CD4^+^ T cells was assessed by intracellular cytokine staining after stimulation with the indicated antigens (PA, p25). Data are mean +/- sem. Results are representative of two independent experiments.
(PDF)

S9 Fig Phagocytosis of opsonized Mtb by J774 macrophages. J774 macrophages were infected with unencapsulated (uncap) or encapsulated (encap) *M*. *tuberculosis* H37Rv, which were previously opsonized with conjugate (H37Rv) serum (CS), pre-immune mouse serum or untreated at an MOI of 10:1, and CFU counts were obtained 2 h after infection. Data shown are representative of 2 independent and similar experiments (*p < 0.05).
(PDF)
