# Supplementary figures and images for: Enhanced control of Mycobacterium tuberculosis extrapulmonary dissemination in mice by an arabinomannan-protein conjugate vaccine
Source: PLoS Pathog. 2017 Mar 9;13(3):e1006250. doi: 10.1371/journal.ppat.1006250 (PMC5360349; doi:10.1371/journal.ppat.1006250)

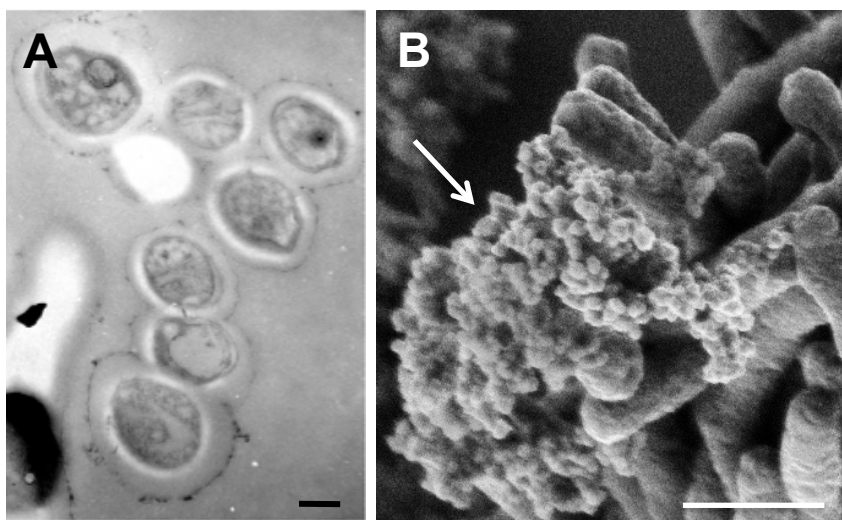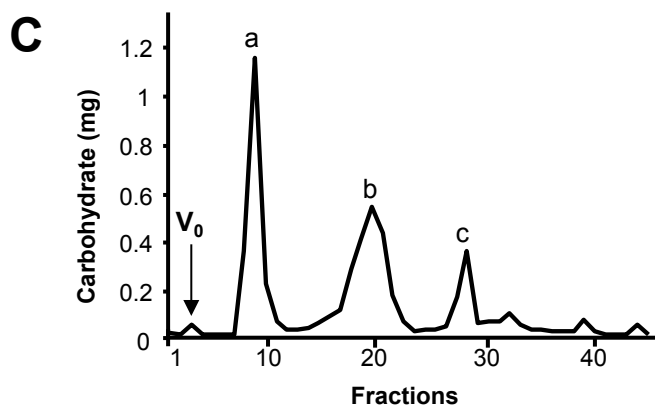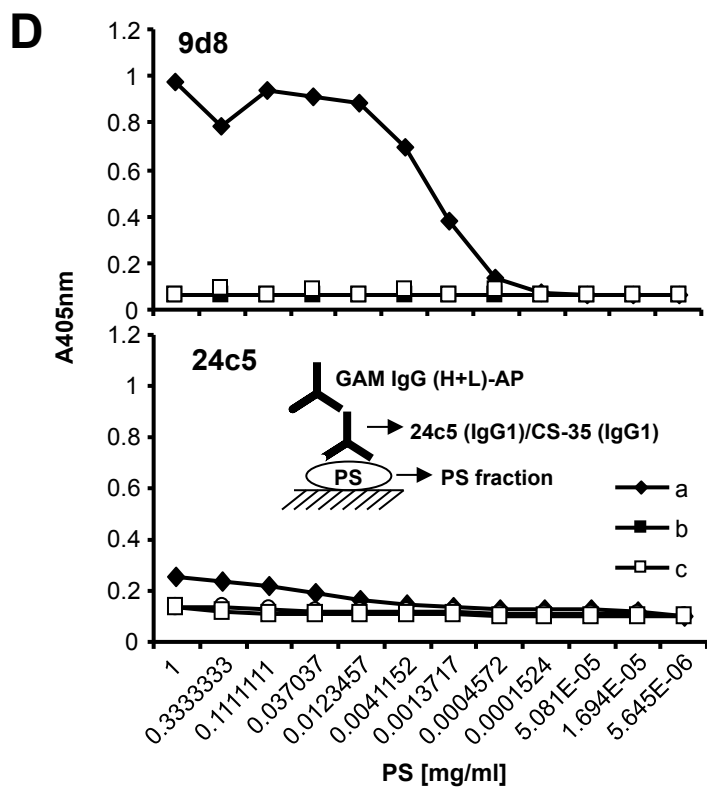

**Supplementary Figure 1**

Supplement: S1 Fig — (A) Electron micrograph of Mtb H37Rv cells grown in minimal media without detergent. Notice the capsule surrounding the cells. Scale bar is 100 nm. (B) Scanning electron micrograph of Mtb H37Rv cells grown in minimal media without detergent. Arrow denotes the polysaccharide capsule. Scale bar 1 μm. (C) Gel chromatography of light Mtb capsular polysaccharides on a PD-10 size exclusion column. Fractions of 4 ml were taken and the carbohydrate content was estimated by phenol-sulphuric acid method. “Vo” means void volume. The pooled fractions are indicated by letters. (D) Binding of 9d8 (anti-AM) (top graph) and 24c5 (anti-α-glucan) (bottom graph) monoclonal antibodies at various concentrations of the indicated PD-10 fractions. The diagram indicates the ELISA configuration. PS, Polysaccharide fraction; AP, alkaline phosphatase; GAM, goat anti-mouse. Capsular polysaccharide isolation was performed up to four times using the same experimental conditions. The results are representative of three independent experiments. (PDF) [file ppat.1006250.s001.pdf]

**A**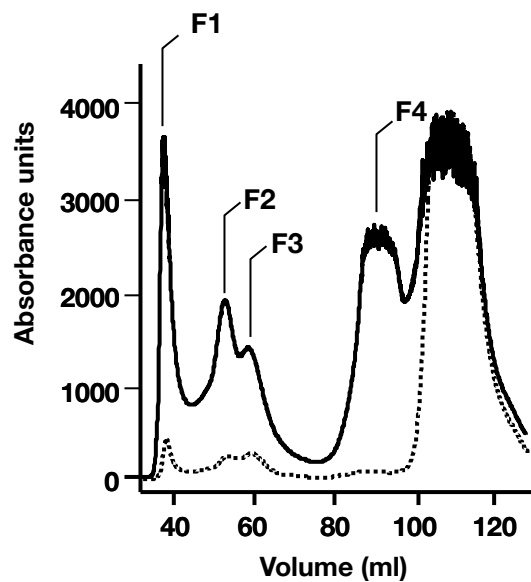**B**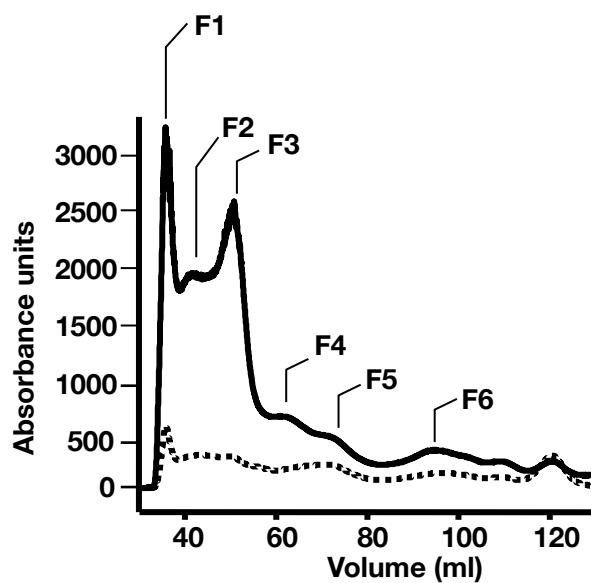

Supplement: S2 Fig — (A,B) Separation of the conjugate reactions AM-Ag85b (A) or AM-PA (B) on Sephacryl S-200 size exclusion column in PBS. Fractions were monitored by on-line measurements of protein content at 280 nm (dotted line) and post-column by measurement of carbohydrate content (straight line) by phenol sulphuric acid method. (PDF) [file ppat.1006250.s002.pdf]

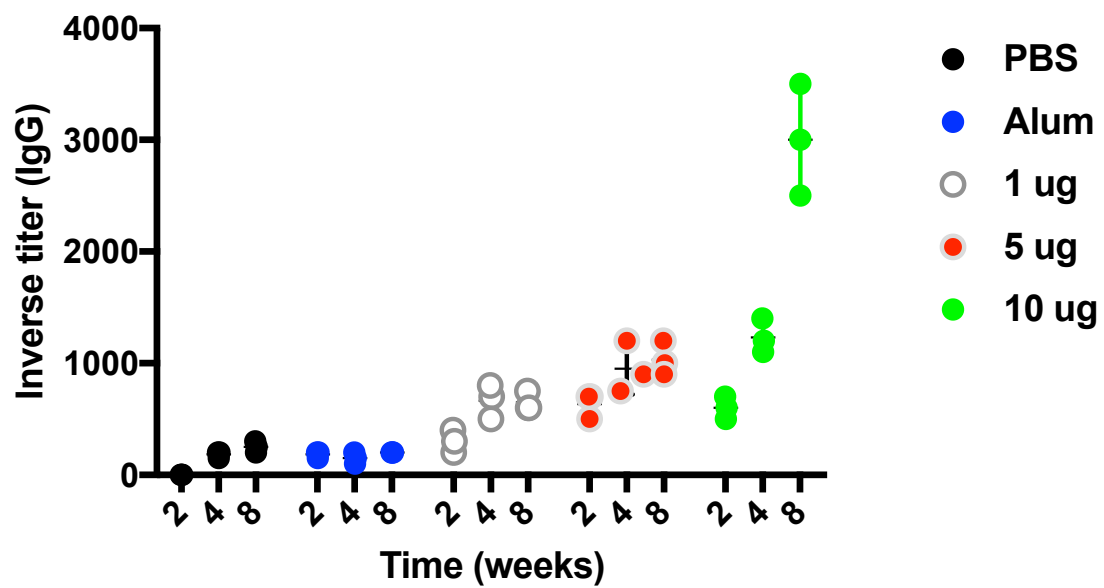

Supplementary Figure 3

Supplement: S3 Fig — Inverse titers (total IgG) of AM-binding antibodies measured by ELISA in serum from C57BL/6 mice (n = 3 per group) immunized with different amounts of AM-Ag85b conjugate. Mice were immunized every two weeks twice after initial immunization. Measurements were performed at 2, 4 and 8 weeks after the initial immunization. (PDF) [file ppat.1006250.s003.pdf]

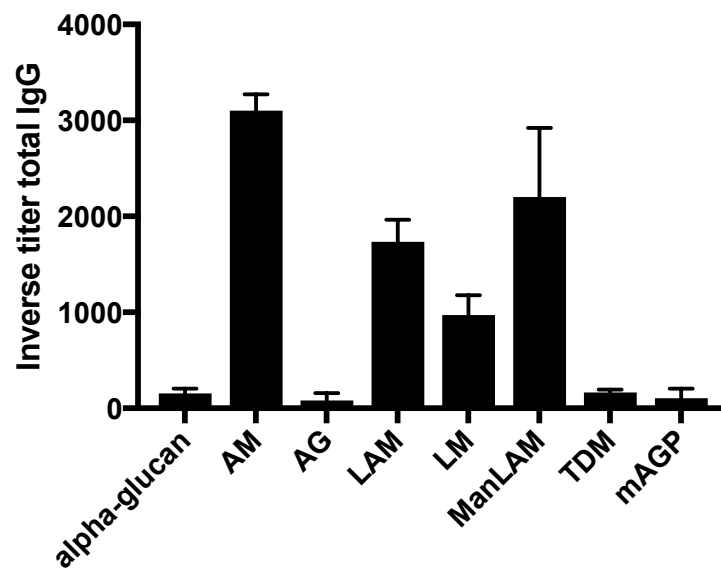

**Supplementary Figure 4**

Supplement: S4 Fig — Inverse titers of Abs from AM-Ag85b conjugate serum for binding to different components of mycobacterial cell surface measured by ELISA in serum from C57BL/6 mice (n = 3 per group). Mice were immunized three times with 10 μg of AM-Ag85b conjugate. The results are representative of three independent experiments performed in the same manner. AM, arabinomannan; AG, arabinogalactan; LAM, lipoarabinomannan; LM, lipomannan; ManLAM, mannose capped LAM; TDM, trehalose deoxy mycolate; mAGP, mycolate arabinogalactan peptidoglycan. complex (PDF) [file ppat.1006250.s004.pdf]

Ag85b-AM MM(1)

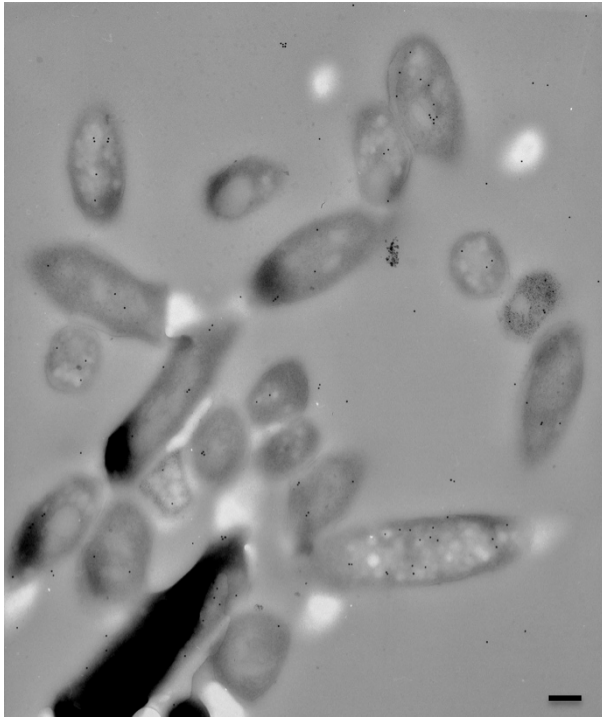

Ag85b-AM MM(2)

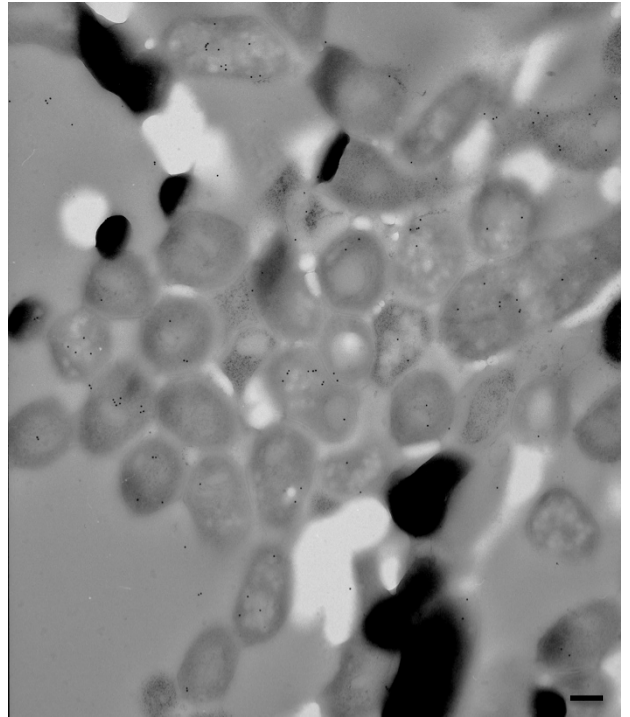

Ag85b-AM MMT(1)

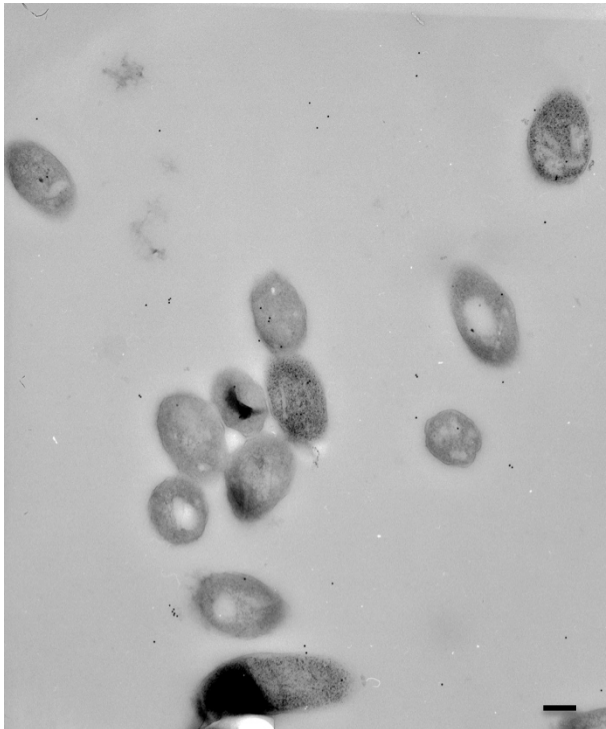

Ag85b-AM MMT (2)

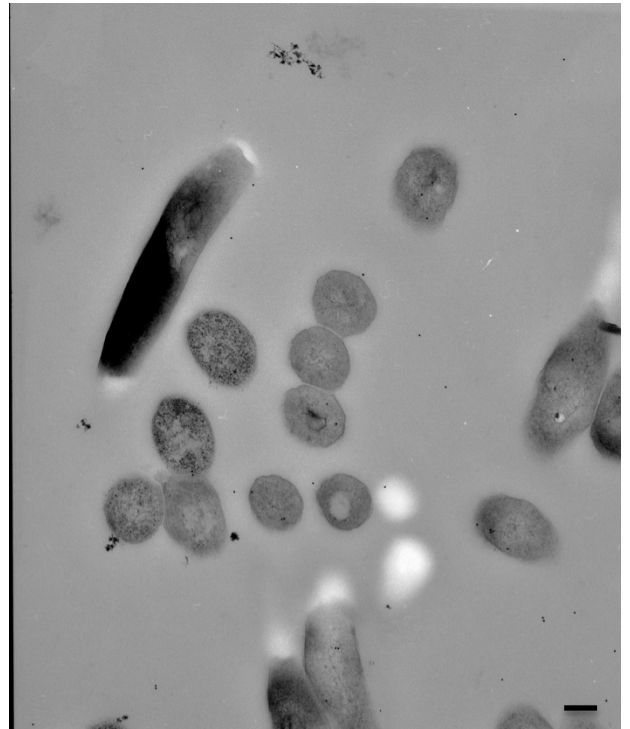

Ag85b-AM MM(1)

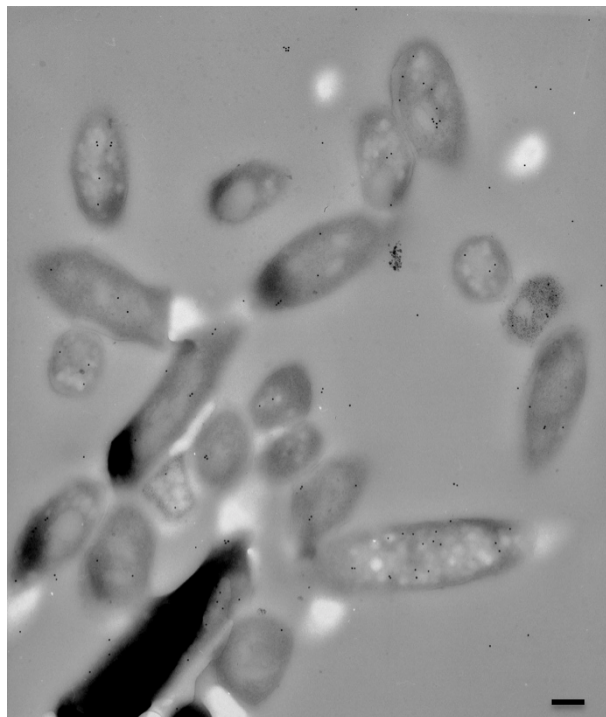

Ag85b-AM MM(2)

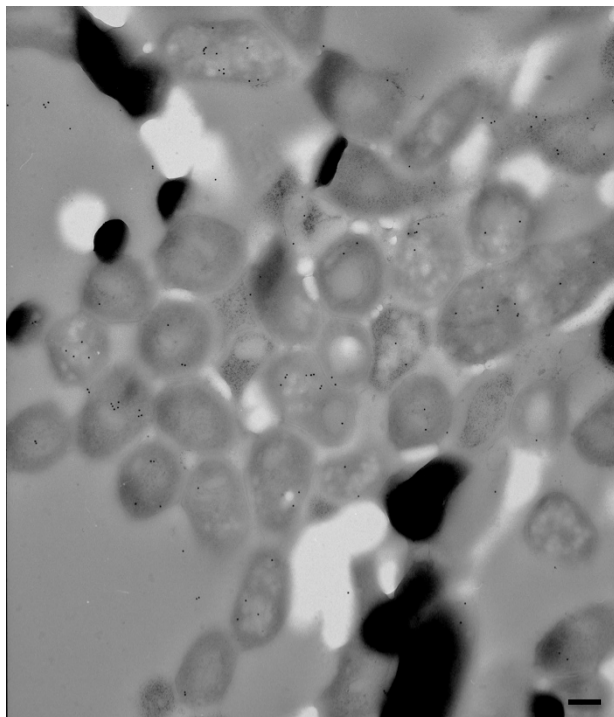

Ag85b-AM MMT(1)

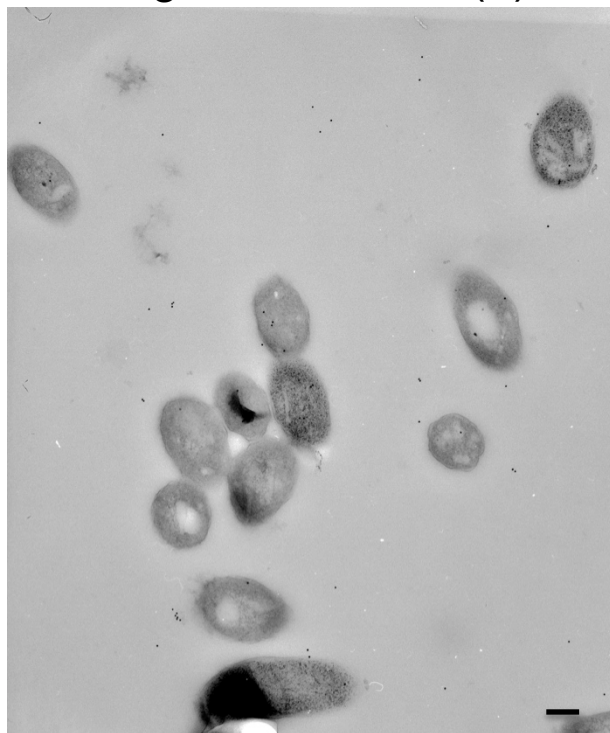

Ag85b-AM MMT (2)

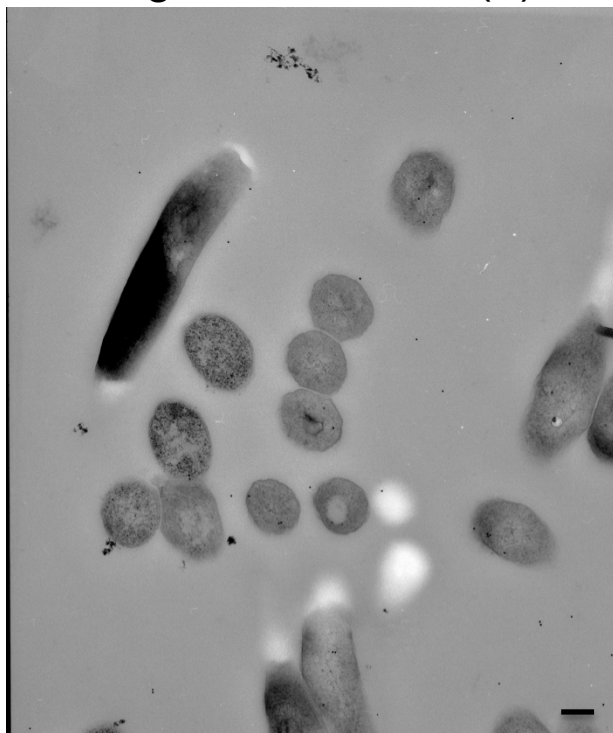

Supplement: S5 Fig — Mtb cells were grown in the presence (MMT) or in the absence of detergent (MM). Scale bar 100 nm. (PDF) [file ppat.1006250.s005.pdf]

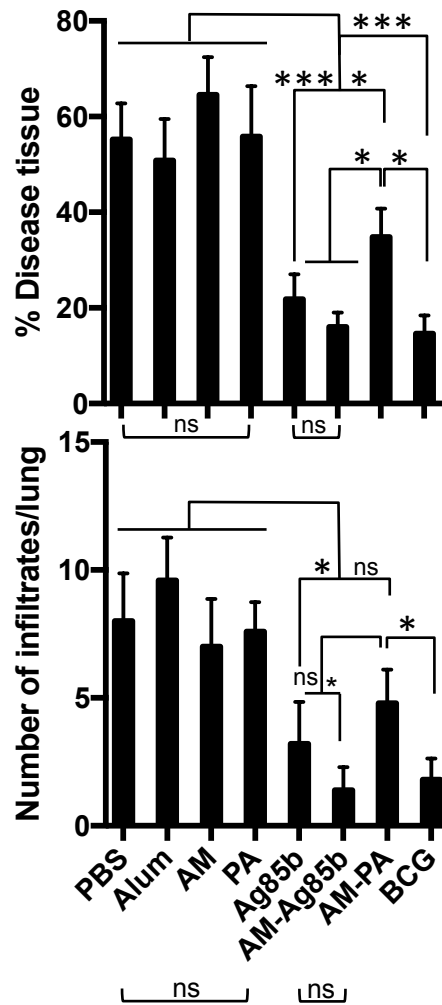

Supplementary Figure 7

Supplement: S7 Fig — (PDF) [file ppat.1006250.s007.pdf]

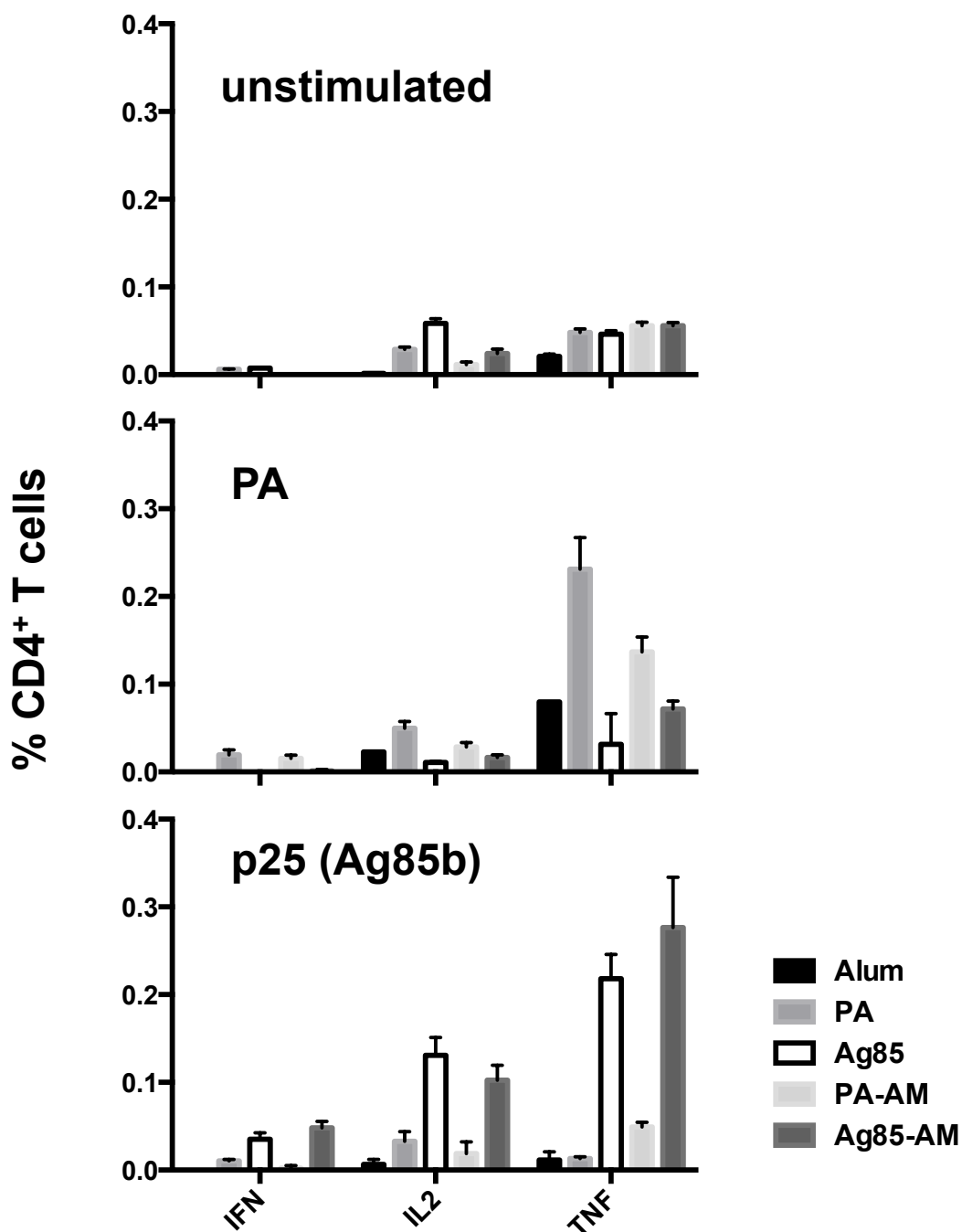

Supplementary Figure 8

Supplement: S8 Fig — Mice were immunized with AM conjugates, PA and Ag85b in Alum and after 4 weeks T cells were isolated. Specificity of CD4+ T cells was assessed by intracellular cytokine staining after stimulation with the indicated antigens (PA, p25). Data are mean +/- sem. Results are representative of two independent experiments. (PDF) [file ppat.1006250.s008.pdf]

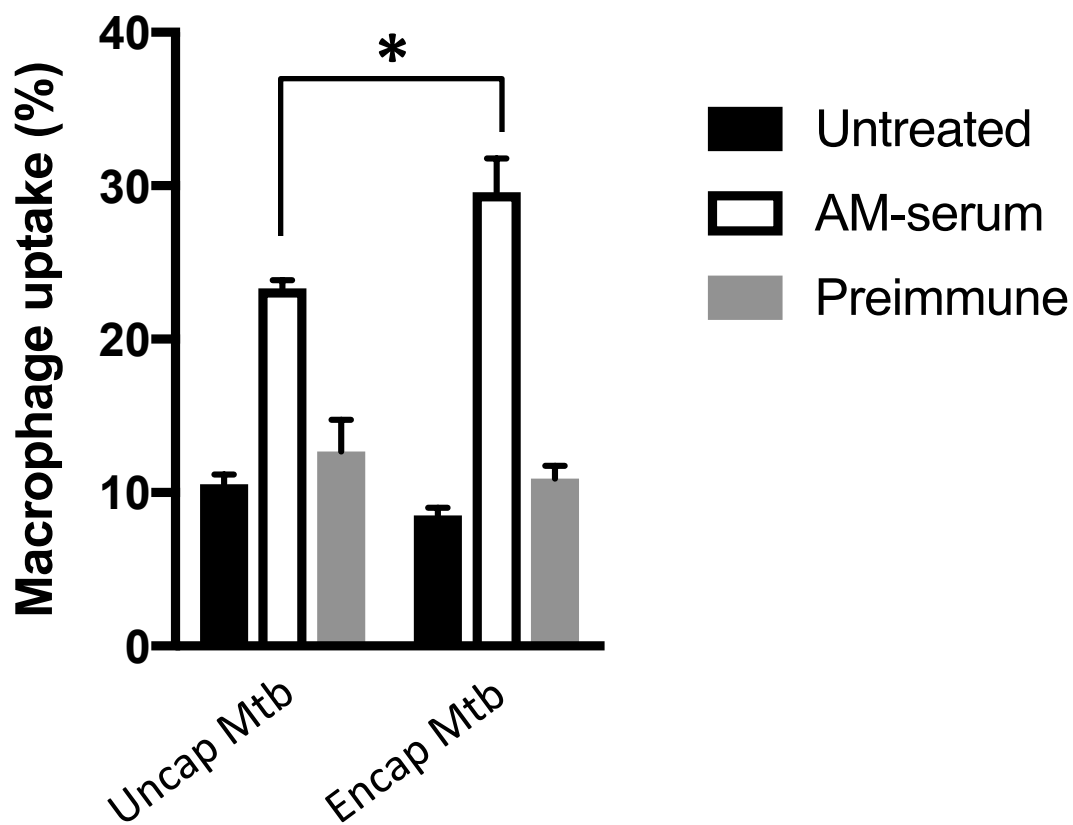

**Supplementary Figure 9**

Supplement: S9 Fig — J774 macrophages were infected with unencapsulated (uncap) or encapsulated (encap) M. tuberculosis H37Rv, which were previously opsonized with conjugate (H37Rv) serum (CS), pre-immune mouse serum or untreated at an MOI of 10:1, and CFU counts were obtained 2 h after infection. Data shown are representative of 2 independent and similar experiments (*p < 0.05). (PDF) [file ppat.1006250.s009.pdf]
