# Supplementary material for: Enhanced control of Mycobacterium tuberculosis extrapulmonary dissemination in mice by an arabinomannan-protein conjugate vaccine
Source: PLoS Pathog. 2017 Mar 9;13(3):e1006250. doi: 10.1371/journal.ppat.1006250 (PMC5360349; doi:10.1371/journal.ppat.1006250)
Supplement: S6 Fig — The symbolic nomenclature used is that recommended by the Consortium for Functional Glycomics. Green circles = mannose; Green stars = arabinose; orange stars = 5-thiomethyl-xylose; white ovals = inositol. The linkage position and stereochemistry between the monosaccharides is indicated over the line connecting them. 3P5 = a phosphate linkage between O3 of the inositol and O5 of an arabinose residue [63]. (PDF) [file ppat.1006250.s006.pdf]

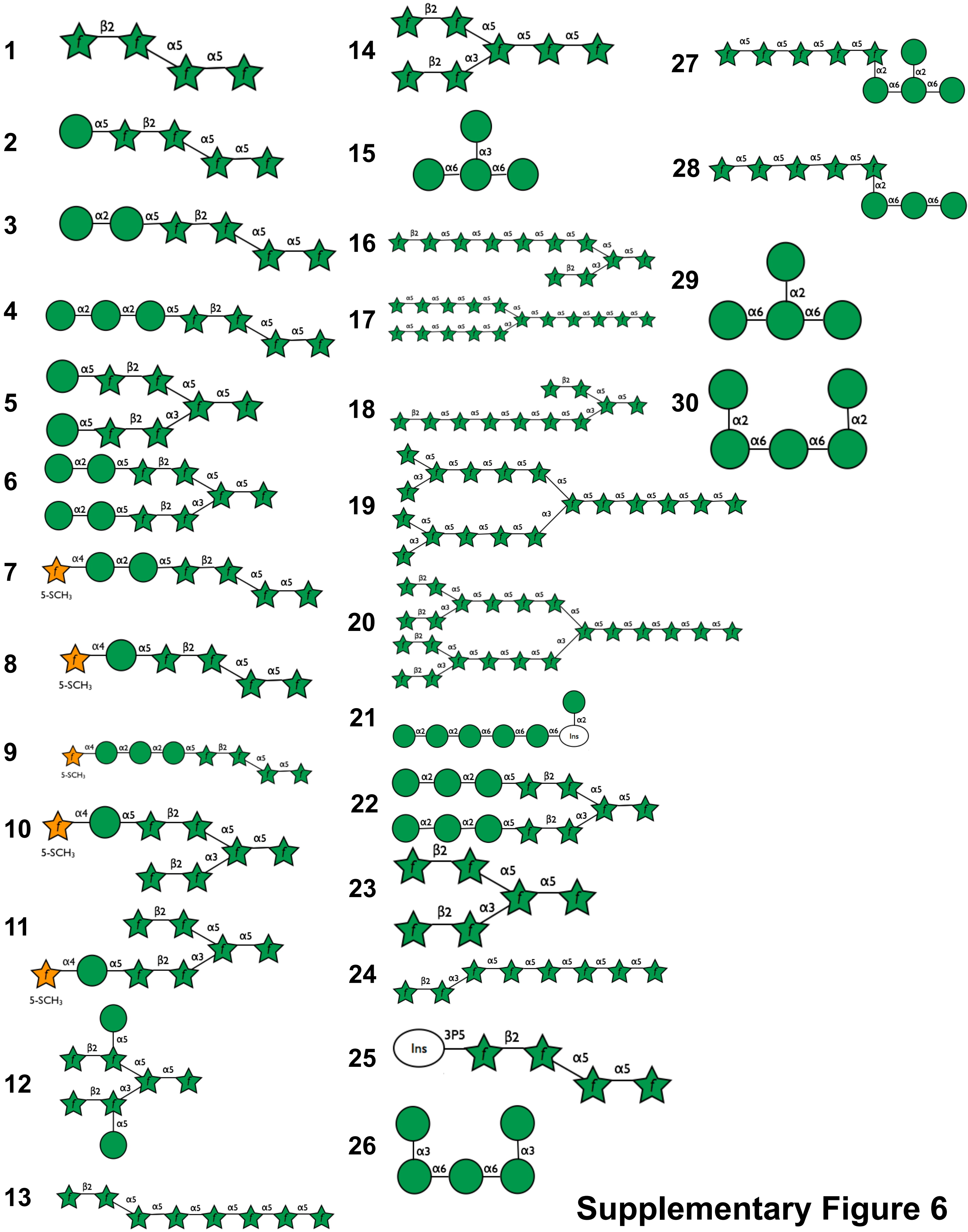

Supplementary Figure 6
